# Newborn and childhood differential DNA methylation and liver fat in school-age children

**DOI:** 10.1186/s13148-019-0799-6

**Published:** 2019-12-31

**Authors:** Madelon L. Geurtsen, Vincent W. V. Jaddoe, Lucas A. Salas, Susana Santos, Janine F. Felix

**Affiliations:** 1 The Generation R Study Group, Erasmus MC, University Medical Center Rotterdam, PO Box 2040, Rotterdam, 3000 CA the Netherlands; 2 Department of Pediatrics, Erasmus MC, University Medical Center Rotterdam, Rotterdam, the Netherlands; 3 Geisel School of Medicine at Dartmouth, Hanover, New Hampshire USA

**Keywords:** Non-alcoholic fatty liver disease, Liver steatosis, Epigenetics, Differentially methylated regions, Magnetic resonance imaging

## Abstract

**Background:**

Non-alcoholic fatty liver disease is the most common chronic liver disease in children in western countries. Adverse early-life exposures are associated with higher liver fat percentages in children. Differential DNA methylation may underlie these associations. We aimed to identify differential DNA methylation in newborns and children associated with liver fat accumulation in childhood. We also examined whether DNA methylation at 22 cytosine-phosphate-guanine sites (CpGs) associated with adult non-alcoholic fatty liver disease is associated with liver fat in children. Within a population-based prospective cohort study, we analyzed epigenome-wide DNA methylation data of 785 newborns and 344 10-year-old children in relation to liver fat fraction at 10 years. DNA methylation was measured using the Infinium HumanMethylation450 BeadChip (Illumina). We measured liver fat fraction by Magnetic Resonance Imaging. Associations of single CpG DNA methylation at the two-time points with liver fat accumulation were analyzed using robust linear regression models. We also analyzed differentially methylation regions using the dmrff package. We looked-up associations of 22 known adult CpGs at both ages with liver fat at 10 years.

**Results:**

The median liver fat fraction was 2.0% (95% range 1.3, 5.1). No single CpGs and no differentially methylated regions were associated with liver fat accumulation. None of the 22 known adult CpGs were associated with liver fat in children.

**Conclusions:**

DNA methylation at birth and in childhood was not associated with liver fat accumulation in 10-year-old children in this study. This may be due to modest sample sizes or DNA methylation changes being a consequence rather than a determinant of liver fat.

## Background

Non-alcoholic fatty liver disease is a pathologic excess of ≥ 5% fat in hepatic cells, not caused by alcohol consumption, genetic or metabolic disorders, medication, or viral infections [1]. Due to the high prevalence of obesity, non-alcoholic fatty liver disease has become the most common chronic liver disease in both children and adults in western countries [2–5]. Non-alcoholic fatty liver disease is associated with an adverse cardio-metabolic risk profile in children [3]. In adults, it is associated with cardio-metabolic diseases and hepatocellular carcinoma, and it is a leading indication for liver transplantation [4, 6]. An accumulating body of evidence suggests that adverse exposures in early life contribute to the development of obesity and non-alcoholic fatty liver disease [5, 7].

The mechanisms underlying the observed associations of early-life factors with liver fat in children and adults may include changes in DNA methylation [5, 7]. DNA methylation is an epigenetic mechanism that is highly dynamic in early life and affects the accessibility of DNA for transcription and thereby gene expression [8]. Various adverse early-life factors have been associated with differential DNA methylation [9–12]. Recent studies using liver biopsy samples of adults with non-alcoholic fatty liver disease suggest differential DNA methylation is cross-sectionally associated with non-alcoholic fatty liver disease [8, 13–15]. A meta-analysis of population-based cohorts in adults identified 22 cytosine-phosphate-guanine sites (CpGs) in peripheral blood at which DNA methylation was associated with non-alcoholic fatty liver disease [6].

We hypothesized that differential DNA methylation at birth and in childhood is associated with liver fat accumulation in children. We performed an epigenome-wide association study (EWAS) to assess whether DNA methylation at birth and at age 10 years is associated with liver fat accumulation measured with magnetic resonance imaging (MRI) in 10-year-old children participating in a population-based prospective cohort study. Analyses were focused on both single CpGs and differentially DNA methylated regions (DMRs). As a secondary analysis, we examined if DNA methylation at birth and at age 10 years is associated with higher (> 2%) versus lower (≤ 2%) liver fat accumulation. We also examined whether DNA methylation at the 22 CpGs known to be associated with non-alcoholic fatty liver disease in adults, is also associated with liver fat in children [6].

## Results

### Subject characteristics

The median liver fat fraction was 2.0% for both groups (newborns 95% range 1.3, 4.6, 10-year-old children 95% range 1.3, 5.1)). The prevalence of non-alcoholic fatty liver disease at age 10 years was 2.2% (*n* = 17/785) in the group with DNA methylation data at birth and 2.6% (*n* = 9/344) in the group with DNA methylation data at age 10 years. The baseline characteristics of the study population are presented in Table 1. Non-response analyses comparing singleton children with DNA methylation data, with and without information on liver fat fraction available, showed that participants in the newborn group were slightly more often female and more often overweight, had somewhat older and higher educated mothers, who more often stopped smoking during pregnancy compared to non-participants in the newborn group. In the childhood group, non-response analyses showed that participants were slightly older compared to non-participants (Table 2).

**Table 1:** Subject characteristics

	Newborns	Childhood
	(*n =* 785)	(*n =* 344)
Maternal characteristics		
Age, mean (SD), years	32.1 ± 4.0	32.1 ± 4.0
Prepregnancy body mass index, mean (SD), kg/m^2^	23.2 ± 3.9	23.4 ± 4.0
Parity, *n* (%), nulliparous	477 (60.8%)	205 (59.6%)
Education, *n* (%), higher education	535 (68.2%)	232 (67.4%)
Smoking during pregnancy, *n* (%), continued	94 (12.0%)	43 (12.5%)
Child characteristics		
Gestational age at birth, median (95%), weeks	40.4 (37.0–42.3)	40.3 (36.9–42.4)
Age, mean (SD), years	0	9.8 ± 0.3
Males, *n* (%)	378 (48.2%)	170 (49.4%)
Birth weight, mean (SD), g	3556 ± 505	3578 ± 515
Body mass index at 10 years, mean (SD), kg/m^2^	17.0 ± 2.1	17.1 ± 2.0
Children with		
Underweight, n (%)	62 (7.9)	19 (5.5)
Normal weight, n (%)	637 (81.1)	287 (83.4)
Overweight, n (%)	79 (10.1)	37 (10.8)
Obesity, n (%)	7 (0.9)	1 (0.3)
Liver fat fraction, median (95% range), %	2.0 (1.3–4.6)	2.0 (1.3–5.1)
Prevalence non-alcoholic fatty liver disease, *n* (%)	17 (2.2%)	9 (2.6%)

Values are observed data and represent means ± SD, medians (95% range), or numbers of subjects (valid %)

**Table 2:** Comparison of child characteristics between children included and not included in the analyses

	Newborns participants	Non-participants	*P* value	Children participants	Non-participants	*P* value
	(*n* = 785)	(*n* = 604)		(*n* = 344)	(*n* = 120)	
Maternal characteristics						
Age, mean (SD), years	32.1 ± 4.0	31.3 ± 4.5	<0.01	32.1 ± 4.0	32.5 ± 4.1	0.41
Prepregnancy body mass index, mean (SD), kg/m^2^	23.2 ± 3.9	23.2 ± 3.8	0.95	23.4 ± 4.0	22.7 ± 3.2	0.12
Parity, *n* (%), nulliparous	477 (60.8%)	365 (60.6%)	0.96	205 (59.6%)	73 (61.3%)	0.74
Education, *n* (%), higher education	535 (68.2%)	357 (61.1%)	<0.01	232 (67.4%)	75 (65.2%)	0.86
Smoking during pregnancy, *n* (%), continued	94 (12.0%)	88 (18.0%)	<0.01	43 (12.5%)	8 (9.3%)	0.70
Child characteristics						
Gestational age at birth, median (95%), weeks	40.4 (37.0–42.3)	40.3 (36.3 – 42.4)	0.45	40.3 (36.9 – 42.4)	40.4 (37.6–42.4)	0.63
Age, mean (SD), years	NA	NA	NA	9.8 ± 0.3	9.7 ± 0.3	<0.01
Males, *n* (%)	378 (48.2%)	325 (53.8%)	0.04	170 (49.4%)	61 (50.8%)	0.79
Birth weight, mean (SD), g	3556 ± 505	3528 ± 518	0.31	3578 ± 515	3547 ± 498	0.57
Body mass index, mean (SD), kg/m^2^	17.0 ± 2.1	17.0 ± 2.1	0.77	17.1 ± 2.0	17.2 ± 2.2	0.62
Children with						
Underweight, *n* (%)	62 (7.9)	30 (8.1)	0.12	19 (5.5)	8 (6.7)	0.21
Normal weight, *n* (%)	637 (81.1)	299 (80.6)	0.05	287 (83.4)	97 (80.8)	0.42
Overweight, *n* (%)	79 (10.1)	38 (6.3)	0.01	37 (10.8)	13 (10.8)	0.52
Obesity, *n* (%)	7 (0.9)	4 (0.7)	0.09	1 (0.3)	2 (1.7)	0.11

Values are observed data and represent means ± SD, medians (95% range), or numbers of subjects (valid %). Differences were tested using Student’s *t* tests and Mann-Whitney tests for normally and non-normally distributed variables, respectively and using *χ2*-test for dichotomous variables

### Epigenome-wide association study of childhood liver fat accumulation

We assessed associations of DNA methylation in cord blood and in whole peripheral blood at 10 years with liver fat as a continuous measure in 10-year-old children. In the main models, adjusted for maternal age, education level, early-pregnancy BMI and smoking, gestational age at birth (cord blood analyses) or child age (childhood analyses), child sex, cell-type proportions, and batch, we did not observe any CpGs at birth or at 10 years to be associated with liver fat accumulation at 10 years after Bonferroni (*p* value < 1.0 × 10^−7^) or false-discovery rate (FDR) correction. The Manhattan plots of both EWAS analysis of liver fat accumulation are presented in Additional file 1: Figure S1a and Figure S1b. Additional file 2: Table S1 and Table S2 show the CpGs with *p* values < 1.0 × 10^−4^ for newborns and for 10-year-old children, respectively. We did not identify significantly associated differentially methylated regions associated with liver fat accumulation, nor did we find associations of individual CpG sites with higher versus lower liver fat accumulation. Additional file 3: Table S3 and Table S4 show the differentially methylated regions with *p* values < 1.0 × 10^−4^ for newborns and 10-year-old children, respectively. Additional file 4: Table S5 and Table S6 show the CpGs with *p* values < 1.0 × 10^−4^ for newborns and for 10-year-old children for higher versus lower liver fat, respectively. Results of the basic model and of the model additionally adjusted for childhood body mass index (BMI) were not substantially different from the results in the main model. The mean percent differences in effect estimates between the main model and the basic model, and between the main model and the childhood BMI model in cord blood were 2.5% and 10.9%, respectively. In the child peripheral blood analyses at 10 years, the mean percent differences were 1.6% and 3.9%, respectively. In Additional file 5: Table S7 and Table S8, we show the results of the basic and childhood BMI models for the CpGs probes with *p* values < 1.0 × 10^−4^ identified in the main model.

### Look-up of CpGs associated with adult liver fat

None of the 22 CpGs differentially methylated regions known for their associations with non-alcoholic fatty liver disease in adults were associated with liver fat in children (Bonferroni corrected *p* value cutoff < 0.05/22 = 2.3 × 10^−3^, Table 3). We found no evidence for enrichment of the 22 CpGs among the 18,848 nominally significant CpGs from the cord blood analysis and among the 23,173 nominally significant CpGs from the 10-year-old analysis (Fisher combined probability *p* value = 1.00 in newborns and *p* value = 0.68 in 10-year-old children).

**Table 3:** Associations of 22 adult non-alcoholic fatty liver disease-associated CpGs with liver fat fraction in children

				Newborns			Children		
CpG	Chromosome	Position	Gene	Effect^*^	SE^*^	*P* value	Effect^*^	SE^*^	*P* value
cg09469355	1	2161886	*SKI*	0.002	0.03	0.96	− 0.002	0.07	0.98
cg17901584	1	55353706	*DHCR24*	− 0.005	0.02	0.74	− 0.069	0.04	0.08
cg03725309	1	109757585	*SARS*	− 0.012	0.02	0.45	0.027	0.05	0.56
cg14476101	1	120255992	*PHGDH*	− 0.003	0.02	0.99	0.011	0.04	0.78
cg19693031	1	145441552	*TXNIP*	− 0.011	0.03	0.72	− 0.031	0.04	0.45
cg06690548	4	139162808	*SLC7A11*	− 0.086	0.05	0.08	− 0.027	0.06	0.67
cg05119988	4	166251189	*SC4MOL*	0.003	0.02	0.88	0.003	0.03	0.92
cg03957124	6	37016869	COX6A1P2^**^	0.011	0.02	0.59	0.027	0.05	0.58
cg18120259	6	43894639	LOC100132354^**^	0.024	0.02	0.31	− 0.124	0.05	0.02
cg17501210	6	166970252	*RPS6KA2*	0.086	0.07	0.21	− 0.137	0.08	0.10
cg21429551	7	30635762	*GARS*	0.015	0.02	0.42	0.017	0.03	0.52
cg11376147	11	57261198	*SLC43A1*	− 0.004	0.03	0.89	0.107	0.07	0.11
cg00574958	11	68607622	*CPT1A*	0.028	0.04	0.43	− 0.019	0.08	0.79
cg26894079	11	122954435	*ASAM*	0.004	0.03	0.88	− 0.020	0.04	0.63
cg11024682	17	17730094	*SREBF1*	− 0.023	0.04	0.54	− 0.005	0.07	0.93
cg14020176	17	72764985	*SLC9A3R1*	0.006	0.03	0.84	− 0.007	0.06	0.90
cg19016694	17	80821826	*TBCD*	0.016	0.03	0.55	− 0.062	0.06	0.30
cg15860624	19	3811194	*ZFR2*	0.011	0.02	0.61	0.002	0.05	0.97
cg02711608	19	47287964	*SLC1A5*	− 0.025	0.03	0.44	0.004	0.06	0.95
cg08309687	21	35320596	LINC00649^**^	− 0.004	0.03	0.88	− 0.008	0.04	0.84
cg27243685	21	43642366	*ABCG1*	0.042	0.04	0.32	− 0.023	0.09	0.81
cg06500161	21	43656587	*ABCG1*	0.018	0.03	0.57	0.023	0.05	0.66

^*^Effect estimates represent the change in liver fat fraction (%) per 10% difference in DNA methylation beta and standard error. Associations are adjusted for maternal age, education level, early-pregnancy BMI and smoking, age at birth or child age at measurement, child sex, cell type proportions, and batch. ^*^Gene names added using information from the UCSC Genome Browser build hg19. Other gene names from original paper by Ma et al. 2019. *BMI* body mass index, *n* number, *SE* standard error

### Candidate genes analysis associated with liver fat

We examined if there was an enrichment of CpGs located in regions within a 4 Mb window (± 2 Mb) surrounding the 9 single-nucleotide polymorphisms (SNPs) identified to be associated with non-alcoholic fatty liver disease in adults, among all nominally significant CpGs in our analyses [16, 17]. A total of 7225 CpGs were present in these regions in the newborn dataset and 7244 CpGs in the 10-year-old dataset. In newborns, 299 of these CpGs were nominally significant (*p* value < 0.05). In 10-year-old children, this was the case for 347 CpGs. There was no enrichment for CpGs associated with liver fat accumulation at either age (Fisher combined probability *p* value = 0.47 in newborns and *p* value = 0.86 in 10-year-old children).

### Top CpG probes functions and related biological processes

In an explorative analysis, significantly enriched gene ontology (GO) terms based on the annotated genes of the 32 CpG probes with *p* values < 1.0 × 10^−4^ in cord blood pointed towards processes related to triglyceride, acylglycerol and lipid metabolic processes, digestive tract development, digestive system development, and digestive tract morphogenesis, among others (Additional file 6: Table S9). The same analysis using the 76 CpG probes with *p* values < 1.0 × 10^−4^ in child peripheral blood revealed processes related to cell cycle functions, organ morphogenesis, and development, among others (Additional file 6: Table S10). We did not observe the functional enrichment of Kyoto Encyclopedia of Genes and Genomes (KEGG) terms ((FDR < 0.05). Next to this, we did not observe significant enrichment of DNAse hypersensitivity sites among the CpG probes with *p* values < 1.0 × 10^−4^ (smallest *p* value in cord blood analyses 0.09 and in childhood analyses 0.25).

## Discussion

In the first epigenome-wide association study on liver fat accumulation in children, we did not observe differential DNA methylation in newborns or 10-year-old children related to liver fat accumulation analyzed as a continuous measure or related to higher versus lower liver fat accumulation measured by MRI at age 10 years. Also, DNA methylation at 22 CpGs known to be associated with non-alcoholic fatty liver disease in adults was not associated with liver fat in children.

### Interpretation of main findings

Non-alcoholic fatty liver disease has an increasing prevalence in both children and adults [5, 18]. It is a major risk factor for adverse cardio-metabolic health in children and for cardio-metabolic diseases and liver diseases in adults [3, 4, 6]. Adverse early-life factors have been described to be associated with liver fat development [5, 7]. These associations may be explained by DNA methylation changes in response to these early-life exposures that lead to liver fat development [5, 19].

Among adults, it has been demonstrated that differential DNA methylation is present in liver biopsy samples of adults with non-alcoholic fatty liver disease [8, 13–15, 20]. All these studies used liver histology, the current gold standard for diagnosing non-alcoholic fatty liver disease [2, 5]. As a consequence, these studies are limited by small sample sizes, histologically heterogeneous groups varying in the severity of the non-alcoholic fatty liver disease, older study populations, wide BMI ranges, and having only few or no healthy controls. None of these reports controlled for cell heterogeneity in their analyses. A recent meta-analysis of four multiethnic population-based cohort studies in adults showed that DNA methylation at 22 CpGs in peripheral blood was associated with non-alcoholic fatty liver disease diagnosed with either computed tomography or ultrasound imaging (FDR < 0.05) [6]. In this study, in newborns and 10-year-old children we did not observe differential DNA methylation at single CpGs or differentially methylated regions in cord blood or child peripheral blood in association with MRI diagnosed liver fat accumulation in 10-year-old children. The associations of the 22 CpGs identified in adults could also not be replicated in children [6]. It is possible that small, but potentially biologically important DNA methylation differences may be associated with liver fat accumulation in children. These differences would be difficult to detect in the moderate sample size of the current study. Besides this, the variability in liver fat accumulation in this population of children was relatively small, which may also partly explain the lack of identified associations. In addition, our study population is a relatively lean population. Associations of DNA methylation with liver fat accumulation may be more apparent among higher-risk populations, as observed in adult studies [8, 13–15, 20]. Another possibility is that DNA methylation truly is not associated with liver fat accumulation in children. As has been suggested for phenotypes such as obesity, differential DNA methylation may be mostly a consequence rather than a cause of liver fat accumulation. If that is indeed the case, then the duration of exposure to increased liver fat in this population of 10-year-old children may not have been sufficient to induce differential DNA methylation [21].

The present population-based study is the first to examine the association of differential DNA methylation with liver fat fraction measured with MRI in children. Although the hypothesis of early-life factors contributing to the development of liver fat accumulation through DNA methylation cannot be completely discarded based on this study, we found no evidence to support associations of differential DNA methylation in newborns or children with liver fat accumulation at 10 years. Future studies should investigate in large longitudinal studies the associations of differential DNA methylation with liver fat accumulation in children.

### Methodological considerations

The strengths of this study are the prospective and cross-sectional analyses with information on DNA methylation at two ages. We used a sensitive imaging-based method to enable non-invasive measurement of liver fat [22, 23]. Although our sample size is relatively large for epigenome-wide analyses, it might still be too small to detect more minor effect sizes [8, 13–15]. We identified no Bonferonni or FDR significant associations for differential DNA methylation in cord blood and in child peripheral blood at 10 years to be associated with liver fat accumulation in childhood. Therefore, the pathway analyses based on the annotated genes of the CpG probes with *p* values < 1.0 × 10^−4^ need to be carefully interpreted. Many of the enriched pathways are based on a relatively low number of genes. As such, the results of the pathway analysis should be considered exploratory and need further confirmation. To the best of our knowledge, similar data on DNA methylation and MRI-measured liver fat accumulation in children are not currently available elsewhere. DNA methylation was measured in blood, which may differ from DNA methylation in liver cells. The relatively small number of children with obesity in the included sample indicates a selection towards a lean population that may affect the generalizability of our findings.

## Conclusions

DNA methylation at birth and in childhood was not associated with liver fat accumulation in 10-year-old children in this study. This may be due to modest sample sizes or DNA methylation changes being a consequence rather than a determinant of liver fat. Future studies should investigate in large longitudinal studies the associations and timing of differential DNA methylation with liver phenotypes in children.

## Methods

### Study design

**Fig. 1:**
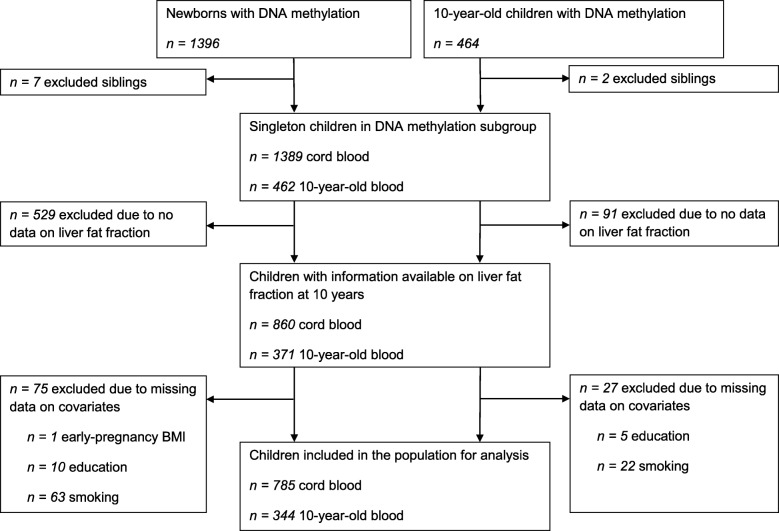
Study participants flow chart

This study was embedded in the Generation R Study, a population-based prospective cohort from early fetal life onwards, based in Rotterdam, the Netherlands [24]. The study has been approved by the Medical Ethical Committee of the Erasmus MC, University Medical Center Rotterdam (MEC 198.782/2001/31). Written informed consent was obtained for all participants [24]. All 9778 participating live-born children were born between April 2002 and January 2006. DNA methylation was measured in a randomly selected European-ancestry subset of 1396 newborns and 464 10-year-old children. The liver fat MRI measurements were performed in a subgroup of children at age 10 years. We excluded children without complete data on liver fat fraction and covariates. The population for analysis of this study comprised 785 newborns and 344 10-year-old children (Fig. 1).

### DNA methylation

DNA was extracted from cord blood and whole peripheral blood at 10 years using the salting-out method. Five hundred nanograms of DNA per sample underwent bisulfite conversion using the EZ-96 DNA Methylation kit (Shallow) (Zymo Research Corporation, Irvine, CA, USA). Samples were plated randomly onto 96-well plates. Samples were processed with the Illumina Infinium HumanMethylation450 (450k) BeadChip (Illumina Inc., San Diego, CA, USA). Quality control of analyzed samples was performed using standardized criteria. Quality control and normalization of the HumanMethylation450 BeadChip array data was performed according to the Control Probe Adjustment and reduction of global CORrelation (CPACOR) workflow using R [25, 26]. Probes that had a detection *p* value ≥ 1E− 16 were set to missing per array. Next, the intensity values were quantile normalized for each of the six probe-type categories separately: type II red/green, type I methylated red/green, and type I unmethylated red/green. Beta values were calculated as the proportion of methylated intensity value to the sum of methylated and unmethylated intensities plus 100. Arrays with observed technical problems such as failed bisulfite conversion, hybridization or extension, as well as arrays with a sex mismatch were removed from subsequent analyses. Additionally, only arrays with a call rate > 95% per sample were processed further. Probes on the X and Y chromosomes were excluded from the analyses. The final datasets contained 457,774 probes in the newborn dataset and 458,563 probes in the 10-year-old dataset. For all CpGs and differentially methylated regions, the official gene name of the nearest gene was noted using Illumina’s annotation information and we enhanced the annotation provided by Illumina with the UCSC Genome Browser build hg19 [27, 28].

### Liver fat fraction at 10 years

We measured liver fat using a 3.0 Tesla MRI (Discovery MR750w, GE Healthcare, Milwaukee, WI, USA) [1, 22–24]. The children wore light clothing without metal objects while undergoing the body scan. A liver fat scan was performed using a single-breath-hold, 3D volume and a special 3-point proton density-weighted Dixon technique (IDEAL IQ) for generating a precise liver fat fraction image [29]. The IDEAL IQ scan is based on a carefully tuned 6-echo echo-planar imaging acquisition. The obtained fat-fraction maps were subsequently analyzed by the Precision Image Analysis (PIA, Kirkland, WA, USA) using the sliceOmatic (TomoVision, Magog, QC, CAN) software package. All extraneous structures and any image artifacts were removed manually [30]. The liver fat fraction was measured independent of any outcome, determined by taking four samples of at least 4 cm^2^ from the central portion of the hepatic volume. Subsequently, the mean signal intensities were averaged to generate an overall mean liver fat fraction estimation. Liver fat fraction measured with IDEAL IQ using MRI is reproducible, highly precise, and validated in adults [31, 32]. As previously described, non-alcoholic fatty liver disease was defined as liver fat fraction ≥ 5.0% [1, 32, 33]. We studied liver fat accumulation across the full spectrum as our primary objective. As the secondary objective, we dichotomized liver fat into low, ≤ 2.0%, and high, > 2.0%, liver fat accumulation. This cutoff was based on the median in our population and on previous work from our group describing that liver fat accumulation above 2.0% is already associated with an increased cardio-metabolic risk profile in children [34]. Due to the lower numbers of cases, we could not dichotomize liver fat accumulation based on the clinical cutoff of ≥ 5.0%.

### Covariates

At enrolment in the study, information on maternal age and educational level was obtained by questionnaires. Maternal smoking during pregnancy was assessed by questionnaires in pregnancy. We measured maternal height and weight at enrolment to calculate early-pregnancy BMI [35]. Information on gestational age at birth, child sex, and age at 10 years visit was obtained from medical records. We measured height and weight in the children, without shoes and heavy clothing. Childhood BMI was calculated and sex- and age-adjusted childhood BMI standard deviation scores were calculated (Growth Analyzer 4.0, Dutch Growth Research Foundation) [36].

### Look-up study of adult CpGs associated with liver fat

We examined in our data the associations of the 22 CpGs known from previous literature to be associated with liver fat accumulation in adults with liver fat accumulation in children [6]. A Bonferroni corrected *p* value < 0.05/22 = 2.3 × 10^−3^ was used to define significance. We also evaluated whether the 22 CpGs were enriched among CpGs with a *p* < 0.05 in our results using a hypergeometric test.

### Genes previously associated with liver fat

We assessed the number of nominally significant single CpGs from our analyses that were located within a 4 Mb window (± 2 Mb) surrounding the 9 SNPs identified in 2 previous genome-wide association studies (GWAS) of liver fat accumulation in adolescents and adults of European descent [16, 17]. With a hypergeometric test, we calculated enrichment of the CpGs surrounding the 9 SNPs among CpGs with a *p* < 0.05 in our results.

### Pathway analysis

To identify biological processes associated with the genes annotated to the CpG probes with *p* values < 1.0 × 10^−4^ identified in cord blood and in child peripheral blood at 10 years associated with liver fat accumulation, we used the DAVID version 6.8 released October 2016 bioinformatics resource to test for enrichment in GO biological processes and KEGG pathways [37]. The online program epigenetic Functional element Overlap analysis of the Results of Genome-Wide Association Study Experiments (eFORGE) was used to examine enrichment for DNAse hypersensitivity site enrichment among the most significantly associated CpGs in both cord blood and in child peripheral blood at 10 years [38].

### Statistical analysis

First, non-response analysis was conducted among singleton children with DNA methylation data, and with or without complete data on liver fat and covariates available, using Student’s *t* tests, Mann-Whitney tests, and chi-square tests. Second, we used robust linear regression models to assess the associations of DNA methylation in cord blood and in whole peripheral blood at 10 years with liver fat fraction as a continuous measure in 10-year-old children [26]. The analyses were performed in three models, namely, a basic model (adjusted for gestational age at birth, child sex, cell type proportions, and batch), a main model (additionally adjusted for maternal age, education level, early-pregnancy BMI, and smoking), and a childhood BMI model (additionally adjusted for childhood BMI at 10 years). The statistical models for DNA methylation measured in 10-year-old children were the same, with the only difference that they were adjusted for child age at the time of measurement instead of gestational age at birth. We adjusted for leukocyte subtypes using the cord blood-specific Gervin reference for the cord blood analyses and the Reinius reference set for the analyses at 10 years using the minfi Bioconductor package in R [39–42]. Included covariates were based on previous studies and strong correlations with DNA methylation and liver fat [2, 6]. Since the outcome of liver fat had a skewed distribution, it was natural log-transformed. Multiple testing was accounted for using Bonferroni correction, with CpGs with a *p* value < 1.0 × 10^−7^ considered significant. Additionally, we planned to report results using FDR correction for multiple testing, using the method by Benjamini and Hochberg [43]. Third, we identified differentially methylated regions using the dmrff package (https://github.com/perishky/dmrff), which identifies differentially methylated regions by combining EWAS summary statistics from nearby CpGs [44]. Significant differentially methylated regions were defined based on the following criteria: (1) within one differentially methylated region, the distance between two neighboring probes can be at most 500 base pairs; (2) the regions have nominal EWAS *p* values < 0.05, and (3) EWAS effect estimates for the individual CpGs in a differentially methylated regions have the same direction. All analyses were performed using R version 3.4.3 [26]. All authors had access to the study data and reviewed and approved the final manuscript.

## Supplementary information

**Additional file 1: Figure S1a.** Epigenome-wide Association Study Results of DNA Methylation in Cord Blood with Liver Fat Fraction in Children. **Figure S1b.** Epigenome-wide Association Study Results of DNA Methylation in Child Peripheral Blood with Liver Fat Fraction in Children.

**Additional file 2: Table S1.** CpG with p-values <1.0 × 10^-4^ from Epigenome-wide Association Study of DNA Methylation in Cord Blood and Child Liver Fat Accumulation in Childhood^*^. **Table S2.** CpG with p-values <1.0 × 10^-4^ from Epigenome-wide Association Study of DNA Methylation in Child Peripheral Blood with Liver Fat Accumulation in Childhood^*^.

**Additional file 3: Table S3.** Differentially Methylated Regions with p-values <1.0 × 10^-4^ of DNA Methylation in Cord Blood with Liver Fat Accumulation in Childhood^*^. **Table S4.** Differentially Methylated Regions with p-values <1.0 × 10^-4^ of DNA Methylation in Child Peripheral Blood with Liver Fat Accumulation in Childhood^*^.

**Additional file 4: Table S5.** CpGs with p-values <1.0 × 10^-4^ from Epigenome-wide Association Study of DNA Methylation in Cord Blood with Higher versus Lower Liver Fat Accumulation in Childhood^*^. **Table S6.** CpGs with p-values <1.0 × 10^-4^ from Epigenome-wide Association Study of DNA Methylation in Child Peripheral Blood with Higher versus Lower Liver Fat Accumulation in Childhood^*^.

**Additional file 5: Table S7.** Results of Basic Model and Child BMI Model for CpGs with p-values <1.0 × 10^-4^ in Main Model in Cord Blood^*^. **Table S8.** Results of Basic Model and Child BMI Model for CpGs with p-values <1.0 × 10^-4^ in Main Model in Child Peripheral Blood^*^.

**Additional file 6: Table S9.** Significantly enriched GO terms among CpGs with p-values <1.0 × 10^-4^ Identified in Cord Blood Associated with Liver Fat Accumulation in Childhood. **Table S10.** Significantly enriched GO terms among CpGs with p-values <1.0 × 10^-4^ Identified in Child Peripheral Blood Associated with Liver Fat Accumulation in Childhood.

## Data Availability

The datasets used and/or analyzed during the current study are available from the corresponding author on reasonable request.

## Abbreviations

BMI: Body mass index
CpGs: Cytosine-phosphate-Guanine sites
eFORGE: Epigenetic Functional element Overlap analysis of the Results of Genome-Wide Association Study Experiments
EWAS: Epigenome-wide association study
FDR: False-discovery rate
GO: Gene ontology
KEGG: Kyoto Encyclopedia of Genes and Genomes
MRI: Magnetic Resonance Imaging
SNPs: Single-nucleotide polymorphisms
