# Supplementary material for: Newborn and childhood differential DNA methylation and liver fat in school-age children
Source: Clin Epigenetics. 2019 Dec 31;12:3. doi: 10.1186/s13148-019-0799-6 (PMC6938624; doi:10.1186/s13148-019-0799-6)
Supplement: Supplementary file 1 — Additional file 1: Figure S1a. Epigenome-wide Association Study Results of DNA Methylation in Cord Blood with Liver Fat Fraction in Children. Figure S1b. Epigenome-wide Association Study Results of DNA Methylation in Child Peripheral Blood with Liver Fat Fraction in Children. [file 13148_2019_799_MOESM1_ESM.docx]

# Figure S1a Epigenome-wide Association Study Results of DNA Methylation in Cord Blood with Liver Fat Fraction in ChildrenManhattan plot of the results of the epigenome-wide association study of DNA methylation in cord blood and liver fat fraction in 10-year-old children (n = 785). The x-axis represents the autosomal (1 – 22) chromosomes and the y-axis shows the –log_10_(p-value). The model was adjusted for maternal age, education level, pre-pregnancy BMI and smoking, gestational age at birth, child sex, cell type proportions and batch.

# Figure 1b Epigenome-wide Association Study Results of DNA Methylation in Child Peripheral Blood with Liver Fat Fraction in Children

Manhattan plot of the results of the epigenome wide association study of DNA methylation in child peripheral blood and liver fat fraction in 10-year-old children (n = 344). The x-axis represents the autosomal (1 – 22) chromosomes and the y-axis shows the –log_10_(p-value). The model was adjusted for maternal age, education level, prepregnancy BMI and smoking, child age at measurement, child sex, cell type proportions and batch.
