# Supplementary material for: Newborn and childhood differential DNA methylation and liver fat in school-age children
Source: Clin Epigenetics. 2019 Dec 31;12:3. doi: 10.1186/s13148-019-0799-6 (PMC6938624; doi:10.1186/s13148-019-0799-6)
Supplement: Supplementary file 2 — Additional file 2: Table S1. CpG with p-values <1.0 × 10^-4^ from Epigenome-wide Association Study of DNA Methylation in Cord Blood and Child Liver Fat Accumulation in Childhood*. Table S2. CpG with p-values <1.0 × 10^-4^ from Epigenome-wide Association Study of DNA Methylation in Child Peripheral Blood with Liver Fat Accumulation in Childhood*. [file 13148_2019_799_MOESM2_ESM.docx]

**Table S1 CpG with p-values <1.0 x 10^-4^ from Epigenome-wide Association Study of DNA Methylation in Cord Blood and Child Liver Fat Accumulation in Childhood^*^**

| CpG | Chromosome | Position | Gene | Effect | SE | P-value |
| --- | --- | --- | --- | --- | --- | --- |
| cg05527491 | 16 | 67184945 | *B3GNT9* | 0.39 | 0.08 | 3.31 x 10^-6^ |
| cg16530382 | 17 | 37123671 | *FBXO47* | 0.05 | 0.01 | 5.59 x 10^-6^ |
| cg08287265 | 10 | 101292766 | *NKX2-3* | -0.34 | 0.08 | 8.94 x 10^-6^ |
| cg24718356 | 6 | 32822171 | *PSMB9, TAP1* | -0.65 | 0.15 | 9.41 x 10^-6^ |
| cg08570275 | 3 | 152553089 | *P2RY1* | 0.34 | 0.08 | 9.65 x 10^-6^ |
| cg01396580 | 1 | 52832012 | *CC2D1B* | 0.81 | 0.18 | 1.19 x 10^-5^ |
| cg12704462 | 12 | 120151527 | *MIR1178, CIT* | 0.28 | 0.06 | 1.58 x 10^-5^ |
| cg15033034 | 3 | 194106984 | *GP5* | 0.11 | 0.03 | 1.66 x 10^-5^ |
| cg18841298 | 12 | 101604258 | *SLC5A8* | -0.09 | 0.02 | 1.70 x 10^-5^ |
| cg13987088 | 9 | 140647870 | *EHMT1* | 0.16 | 0.04 | 1.72 x 10^-5^ |
| cg13223721 | 7 | 131720532 | *AC105443.2* | -0.21 | 0.05 | 1.83 x 10^-5^ |
| cg05551922 | 3 | 49396232 | *GPX1* | -0.20 | 0.05 | 1.92 x 10^-5^ |
| cg00113096 | 2 | 134119805 | *NCKAP5* | -0.09 | 0.02 | 2.04 x 10^-5^ |
| cg10368518 | 4 | 71859343 | *DCK* | -0.48 | 0.11 | 2.23 x 10^-5^ |
| cg16530177 | 6 | 6320324 | *F13A1* | -0.09 | 0.02 | 2.75 x 10^-5^ |
| cg23915314 | 14 | 24550845 | *NRL* | -0.50 | 0.12 | 2.99 x 10^-5^ |
| cg17492481 | 5 | 134735068 | *MACROH2A1* | -0.67 | 0.16 | 3.25 x 10^-5^ |
| cg12732390 | 3 | 79189311 | *ROBO1* | -0.10 | 0.02 | 4.66 x 10^-5^ |
| cg24673605 | 3 | 46890144 | *MYL3* | 0.13 | 0.03 | 4.69 x 10^-5^ |
| cg09274253 | 16 | 896724 | *LMF1* | 0.22 | 0.06 | 4.78 x 10^-5^ |
| cg08882503 | 1 | 5959093 | *NPHP4* | 0.21 | 0.05 | 5.16 x 10^-5^ |
| cg18554922 | 14 | 104870666 | *RNU6-684P* | 0.22 | 0.05 | 5.31 x 10^-5^ |
| cg27065979 | 13 | 52734134 | *NEK3* | -0.27 | 0.07 | 5.37 x 10^-5^ |
| cg19817544 | 15 | 37109747 | *CSNK1A1P1* | -0.16 | 0.04 | 5.58 x 10^-5^ |
| cg25751266 | 3 | 38495385 | *ACVR2B-AS1, ACVR2B* | -0.45 | 0.11 | 5.86 x 10^-5^ |
| cg02397368 | 5 | 2564206 | *RP11-129I19.2* | -0.12 | 0.03 | 6.34 x 10^-5^ |
| cg23042796 | 2 | 89065350 | *ANKRD36BP2* | -0.33 | 0.08 | 6.91 x 10^-5^ |
| cg16315928 | 6 | 109776240 | *MICAL1* | -0.56 | 0.14 | 7.35 x 10^-5^ |
| cg21847720 | 8 | 2075777 | *MYOM2* | 0.02 | 0.01 | 8.10 x 10^-5^ |
| cg02063915 | 11 | 119600090 | *VECTIN1* | 0.35 | 0.09 | 8.25 x 10^-5^ |
| cg16052901 | 6 | 33240864 | *VPS52, RPS18* | 0.11 | 0.03 | 8.42 x 10^-5^ |
| cg18296281 | 11 | 9286584 | *DENND5A* | 0.99 | 0.25 | 9.04 x 10^-5^ |

^*^Effect estimates represent the change in liver fat fraction (%) per 10% difference in DNA methylation beta and standard error in newborns. Associations are adjusted for maternal age, education level, early-pregnancy BMI and smoking, gestational age at birth, child sex, cell type proportions and batch. BMI, Body Mass Index, n, number, SE, standard error.

**Table S2 CpG with p-values <1.0 x 10^-4^ from Epigenome-wide Association Study of DNA Methylation in Child Peripheral Blood with Liver Fat Accumulation in Childhood^*^**

| CpG | Chromosome | Position | Gene | Effect | SE | P-value |
| --- | --- | --- | --- | --- | --- | --- |
| cg12020444 | 12 | 130821456 | *PIWIL1* | 0.30 | 0.06 | 1.54 x 10^-7^ |
| cg00946960 | 10 | 14470897 | *FRMD4A* | 0.15 | 0.03 | 1.44 x 10^-6^ |
| cg10573751 | 7 | 27144391 | *HOXA3* | -0.16 | 0.03 | 1.59 x 10^-6^ |
| cg19497388 | 20 | 25040831 | *ACSS1* | -0.41 | 0.09 | 4.96 x 10^-6^ |
| cg00549910 | 2 | 80531597 | *CTNNA2, LRRTM1* | -0.74 | 0.16 | 5.78 x 10^-6^ |
| cg13571972 | 9 | 132177348 | *RP11-65J3.3* | -1.48 | 0.33 | 6.91 x 10^-6^ |
| cg05837235 | 17 | 77043815 | *C1QTNF1* | 0.69 | 0.15 | 7.70 x 10^-6^ |
| cg16555595 | 13 | 114201796 | *TMCO3* | 0.45 | 0.11 | 7.75 x 10^-6^ |
| cg17051207 | 12 | 49482508 | *DHH* | 0.22 | 0.05 | 8.26 x 10^-6^ |
| cg00378658 | 11 | 1232347 | *MUC5AC* | 0.15 | 0.33 | 8.35 x 10^-6^ |
| cg01871127 | 2 | 177026301 | *HOXD3* | 0.40 | 0.09 | 8.47 x 10^-6^ |
| cg01282508 | 19 | 41319790 | *CYP2T1P* | -0.04 | 0.01 | 8.85 x 10^-6^ |
| cg23763836 | 10 | 125769668 | *CHST15* | 0.73 | 0.17 | 9.70 x 10^-6^ |
| cg19398365 | 13 | 113500667 | *ATP11A* | -0.39 | 0.09 | 1.01 x 10^-5^ |
| cg18471160 | 1 | 201083459 | *ASCL5* | -0.54 | 0.12 | 1.18 x 10^-5^ |
| cg20489847 | 8 | 144095483 | *LY6E-DT* | -0.42 | 0.10 | 1.19 x 10^-5^ |
| cg18857369 | 14 | 105518248 | *GPR132* | -0.31 | 0.07 | 1.21 x 10^-5^ |
| cg05511958 | 2 | 113342738 | *CHCHD5* | -0.32 | 0.75 | 1.35 x 10^-5^ |
| cg09071239 | 20 | 23336944 | *LINC01431* | -0.35 | 0.08 | 1.36 x 10^-5^ |
| cg00167491 | 5 | 44388799 | *FGF10* | -0.46 | 0.11 | 1.86 x 10^-5^ |
| cg02110701 | 16 | 88556838 | *ZFPM1* | -0.83 | 0.19 | 1.87 x 10^-5^ |
| cg13680337 | 10 | 71626580 | *COL13A1* | 0.15 | 0.04 | 1.97 x 10^-5^ |
| cg20896197 | 10 | 91461107 | *KIF20B* | -0.82 | 0.19 | 2.14 x 10^-5^ |
| cg26579556 | 14 | 103673450 | *RP11-736N17.9* | 0.12 | 0.03 | 2.18 x 10^-5^ |
| cg26886411 | 13 | 55015275 | *RPL13AP25* | -0.40 | 0.09 | 2.19 x 10^-5^ |
| cg08264376 | 21 | 37833864 | *CLDN14* | -0.23 | 0.05 | 2.28 x 10^-5^ |
| cg23303505 | 6 | 29911836 | *HLA-A* | -0.06 | 0.01 | 2.40 x 10^-5^ |
| cg01294686 | 1 | 26606093 | *SH3BGRL3* | -0.33 | 0.08 | 241 x 10^-5^ |
| cg13155079 | 11 | 44286499 | *ALX4* | -0.34 | 0.08 | 2.43 x 10^-5^ |
| cg24407942 | 6 | 170180742 | *ERMARD* | -0.50 | 0.12 | 2.45 x 10^-5^ |
| cg03290827 | 17 | 75180760 | *SEC14L1* | 0.22 | 0.05 | 2.48 x 10^-5^ |
| cg06769094 | 8 | 41151382 | *SFRP1* | 0.67 | 0.16 | 2.49 x 10^-5^ |
| cg15594605 | 4 | 7289763 | *SORCS2* | 0.04 | 0.01 | 2.54 x 10^-5^ |
| cg27107150 | 16 | 1272275 | *TPSG1* | -0.30 | 0.07 | 2.57 x 10^-5^ |
| cg12000458 | 17 | 19192696 | *EPN2* | -0.41 | 0.10 | 2.63 x 10^-5^ |
| cg23055496 | 3 | 6906371 | *GRM7* | 0.20 | 0.05 | 2.63 x 10^-5^ |
| cg16244155 | 18 | 77905565 | *PARD6G-AS1* | -0.10 | 0.02 | 2.97 x 10^-5^ |
| cg19310458 | 2 | 80361612 | *CTNNA2* | 0.25 | 0.06 | 3.04 x 10^-5^ |
| cg12061285 | 19 | 5212165 | *PTPRS* | -0.44 | 0.11 | 3.31 x 10^-5^ |
| cg05251669 | 18 | 77905391 | *PARD6G-AS1* | -0.09 | 0.02 | 3.33 x 10^-5^ |
| cg14694712 | 19 | 43683698 | *PSG5* | 0.16 | 0.04 | 3.53 x 10^-5^ |
| cg10829004 | 14 | 60179825 | *RTN1* | -0.38 | 0.09 | 3.83 x 10^-5^ |
| cg04139359 | 1 | 45085438 | *RNF220* | -0.30 | 0.07 | 3.89 x 10^-5^ |
| cg17515773 | 5 | 57755861 | *PLK2* | -0.46 | 0.11 | 4.11 x 10^-5^ |
| cg25213720 | 5 | 176734343 | *MXD3* | 0.23 | 0.06 | 4.11 x 10^-5^ |
| cg17382048 | 11 | 118754386 | *CXCR5* | 0.20 | 0.05 | 4.20 x 10^-5^ |
| cg25084220 | 5 | 126366197 | *MARCHF3* | 1.59 | 0.39 | 4.28 x 10^-5^ |
| cg01817897 | 5 | 160365430 | *LINC02159* | -0.34 | 0.08 | 4.52 x 10^-5^ |
| cg09321758 | 12 | 54343506 | *HOXC13* | 0.47 | 0.12 | 4.74 x 10^-5^ |
| cg21405786 | 7 | 148990485 | *ZNF783* | -0.16 | 0.04 | 4.84 x 10^-5^ |
| cg14040899 | 10 | 1256651 | *ADARB2* | -0.36 | 0.09 | 5.08 x 10^-5^ |
| cg01031312 | 3 | 195849513 | *LINC00885* | -0.19 | 0.05 | 5.10 x 10^-5^ |
| cg07015412 | 15 | 78025260 | *LINGO1* | -0.09 | 0.02 | 5.17 x 10^-5^ |
| cg26216343 | 10 | 134018934 | *DPYSL4* | -0.05 | 0.12 | 5.18 x 10^-5^ |
| cg17235827 | 6 | 33396215 | *SYNGAP1* | 0.40 | 0.10 | 5.55 x 10^-5^ |
| cg18994015 | 2 | 32386706 | *SPAST* | 0.14 | 0.03 | 6.30 x 10^-5^ |
| cg24957532 | 5 | 118730026 | *TNFAIP8* | -0.24 | 0.06 | 6.42 x 10^-5^ |
| cg04496920 | 16 | 2032394 | *NOXO1* | 0.15 | 0.04 | 6.66 x 10^-5^ |
| cg13657092 | 22 | 26986419 | *TPST2* | 0.46 | 0.12 | 6.70 x 10^-5^ |
| cg19430975 | 8 | 145515787 | *HSF1, BOP1* | 0.67 | 0.17 | 6.93 x 10^-5^ |
| cg16737533 | 18 | 77905355 | *PARD6G-AS1* | -0.11 | 0.03 | 6.96 x 10^-5^ |
| cg02734482 | 3 | 129761854 | *OR7E21P* | -0.26 | 0.06 | 7.06 x 10^-5^ |
| cg12128483 | 7 | 129691109 | *ZC3HC1* | 0.15 | 0.04 | 7.16 x 10^-5^ |
| cg23559165 | 12 | 8598739 | *OR7E149P* | 0.26 | 0.07 | 7.18 x 10^-5^ |
| cg05352250 | 4 | 1245662 | *CTBP1-DT* | -0.39 | 0.10 | 7.22 x 10^-5^ |
| cg13071609 | 3 | 14336117 | *RP11-536I6.2* | -0.31 | 0.78 | 7.28 x 10^-5^ |
| cg16991637 | 1 | 226322955 | *RP11-396C23.3* | -0.16 | 0.04 | 7.48 x 10^-5^ |
| cg04815301 | 10 | 118956023 | *KCNK18* | 0.37 | 0.09 | 7.76 x 10^-5^ |
| cg13985132 | 15 | 65394414 | *UBAP1L* | 0.11 | 0.03 | 7.77 x 10^-5^ |
| cg08759899 | 5 | 101831271 | *SLCO6A1* | -0.38 | 0.10 | 7.91 x 10^-5^ |
| cg03693434 | 17 | 80050880 | *FASN* | -0.33 | 0.08 | 8.20 x 10^-5^ |
| cg21553199 | 8 | 22527157 | *BIN3* | 0.22 | 0.05 | 8.54 x 10^-5^ |
| cg01953119 | 2 | 20425340 | *SDC1* | -0.47 | 0.19 | 8.72 x 10^-5^ |
| cg14315558 | 21 | 26934682 | *MIR155HG* | -0.46 | 0.12 | 9.37 x 10^-5^ |
| cg27033805 | 8 | 145499436 | *BOP1* | -0.09 | 0.02 | 9.59 x 10^-5^ |
| cg10177518 | 15 | 26480873 | *LINC00929* | 0.08 | 0.02 | 9.86 x 10^-5^ |

^*^Effect estimates represent the change in liver fat fraction (%) per 10% difference in DNA methylation beta and standard error in 10-year-old children. Associations are adjusted for maternal age, education level, early-pregnancy BMI and smoking, child age at measurement, child sex, cell type proportions and batch. BMI, Body Mass Index, n, number, SE, standard error.
