# Supplementary material for: Newborn and childhood differential DNA methylation and liver fat in school-age children
Source: Clin Epigenetics. 2019 Dec 31;12:3. doi: 10.1186/s13148-019-0799-6 (PMC6938624; doi:10.1186/s13148-019-0799-6)
Supplement: Supplementary file 3 — Additional file 3: Table S3. Differentially Methylated Regions with p-values <1.0 × 10^-4^ of DNA Methylation in Cord Blood with Liver Fat Accumulation in Childhood*. Table S4. Differentially Methylated Regions with p-values <1.0 × 10^-4^ of DNA Methylation in Child Peripheral Blood with Liver Fat Accumulation in Childhood*. [file 13148_2019_799_MOESM3_ESM.docx]

**Table S3 Differentially Methylated Regions with p-values <1.0 x 10^-4^ of DNA Methylation in Cord Blood with Liver Fat Accumulation in Childhood^*^**

| Chromosome | Start | End | N of probes | Gene | Effect | SE | P-value |
| --- | --- | --- | --- | --- | --- | --- | --- |
| 17 | 37123669 | 37123767 | 4 | *FBXO47* | 0.05 | 0.01 | 3.76 x 10^-6^ |
| 5 | 138729068 | 138729245 | 2 | *PROB1* | 0.22 | 0.05 | 6.57 x 10^-6^ |
| 2 | 97171137 | 97171218 | 2 | *NEURL3* | -0.15 | 0.04 | 2.00 x 10^-5^ |
| 8 | 136245867 | 136246255 | 3 | *LINC01591* | -0.08 | 0.02 | 2.82 x 10^-5^ |
| 6 | 32820577 | 32821155 | 21 | *SEC14L2* | -0.07 | 0.02 | 4.83 x 10^-5^ |
| 13 | 114189945 | 114189998 | 2 | *TMCO3* | 0.21 | 0.05 | 5.30 x 10^-5^ |
| 2 | 114384633 | 114384860 | 3 | *RPL23AP7, RABL2A* | -0.28 | 0.07 | 5.46 x 10^-5^ |
| 12 | 69202458 | 69202640 | 3 | *MDM2* | 0.21 | 0.05 | 5.74 x 10^-5^ |
| 2 | 172951966 | 172952415 | 3 | *DLX1* | 0.10 | 0.03 | 9.48 x 10^-5^ |
| 5 | 180324804 | 180325579 | 3 | *BTNL8* | -0.10 | 0.03 | 9.55 x 10^-5^ |

^*^Results present identified DMRs from association analyses of DNA-methylation in newborns with childhood liver fat fraction (%) per 10% difference in DNA methylation beta and standard error. Associations are adjusted for maternal age, education level, early-pregnancy BMI and smoking, gestational age at birth, child sex, cell type proportions and batch. BMI, Body Mass Index, n, number, SE, standard error.

**Table S4 Differentially Methylated Regions with p-values <1.0 x 10^-4^ of DNA Methylation in Child Peripheral Blood with Liver Fat Accumulation in Childhood^*^**

| Chromosome | Start | End | N of probes | Gene | Effect | SE | P-value |
| --- | --- | --- | --- | --- | --- | --- | --- |
| 7 | 3033579 | 3033636 | 2 | *CARD11* | -0.27 | 0.06 | 1.83 x 10^-6^ |
| 6 | 30130226 | 30130458 | 4 | *TRIM15* | -0.14 | 0.03 | 2.30 x 10^-6^ |
| 7 | 141673384 | 141673649 | 3 | *MGAM* | 0.17 | 0.04 | 4.78 x 10^-6^ |
| 14 | 103673397 | 103673450 | 2 | *RP11-736N17.9* | 0.12 | 0.03 | 4.92 x 10^-6^ |
| 6 | 312058 | 312105 | 2 | *DUSP22* | -0.22 | 0.05 | 5.13 x 10^-6^ |
| 11 | 64879061 | 64879080 | 2 | *AP003068.9* | 0.18 | 0.04 | 6.37 x 10^-6^ |
| 1 | 228593622 | 228593833 | 3 | *TRIM11* | 0.30 | 0.07 | 8.66 x 10^-6^ |
| 22 | 20791733 | 20792222 | 4 | *SCARF2* | 0.31 | 0.07 | 1.33 x 10^-5^ |
| 4 | 2061325 | 2061507 | 2 | *NAT8L* | -0.78 | 0.18 | 1.79 x 10^-5^ |
| 4 | 8477793 | 8477915 | 2 | *TRMT44* | -0.22 | 0.05 | 1.90 x 10^-5^ |
| 17 | 32645753 | 32646006 | 3 | *CCL8* | 0.10 | 0.02 | 1.94 x 10^-5^ |
| 18 | 77905355 | 77905391 | 2 | *ADNP2* | -0.09 | 0.02 | 2.54 x 10^-5^ |
| 14 | 60973773 | 60973823 | 2 | *C14orf39* | 0.30 | 0.07 | 2.84 x 10^-5^ |
| 4 | 53578311 | 53578359 | 2 | *DANCR* | 0.12 | 0.03 | 3.34 x 10^-5^ |
| 15 | 68098945 | 68098991 | 2 | *MAP2K5* | 0.35 | 0.08 | 3.42 x 10^-5^ |
| 6 | 29911334 | 29911339 | 2 | *HLA-A* | -0.09 | 0.02 | 3.46 x 10^-5^ |
| 19 | 590413 | 590538 | 2 | *HCN2, AC005559.3* | -0.53 | 0.13 | 5.67 x 10^-5^ |
| 13 | 95366050 | 95366203 | 2 | *SOX21-AS1* | 0.15 | 0.04 | 5.71 x 10^-5^ |
| 13 | 103053652 | 103054076 | 3 | *FGF14* | 0.10 | 0.03 | 6.09 x 10^-5^ |
| 1 | 16553267 | 16553329 | 3 | *ANO7L1* | 0.17 | 0.04 | 6.79 x 10^-5^ |
| 15 | 65809997 | 65810204 | 7 | *DPP8* | 0.16 | 0.04 | 7.14 x 10^-5^ |
| 8 | 54164310 | 54164374 | 2 | *OPRK1* | 0.31 | 0.08 | 7.16 x 10^-5^ |
| 1 | 119527884 | 119528018 | 2 | *TBX15* | -0.17 | 0.04 | 8.31 x 10^-5^ |
| 11 | 63997586 | 63997668 | 2 | *RP11-783K16.14* | 0.21 | 0.05 | 8.42 x 10^-5^ |
| 6 | 136610914 | 136610989 | 2 | *BCLAF1* | -0.55 | 0.14 | 9.07 x 10^-5^ |
| 6 | 15505345 | 15505949 | 4 | *JARID2* | -0.15 | 0.04 | 9.41 x 10^-5^ |

^*^Results present identified DMRs from association analyses of DNA-methylation in 10-year-old children with childhood liver fat fraction (%) per 10% difference in DNA methylation beta and standard error. Associations are adjusted for maternal age, education level, early-pregnancy BMI and smoking, child age at measurement, child sex, cell type proportions and batch. BMI, Body Mass Index, n, number, SE, standard error.
