# Supplementary material for: Newborn and childhood differential DNA methylation and liver fat in school-age children
Source: Clin Epigenetics. 2019 Dec 31;12:3. doi: 10.1186/s13148-019-0799-6 (PMC6938624; doi:10.1186/s13148-019-0799-6)
Supplement: Supplementary file 4 — Additional file 4: Table S5. CpGs with p-values <1.0 × 10^-4^ from Epigenome-wide Association Study of DNA Methylation in Cord Blood with Higher versus Lower Liver Fat Accumulation in Childhood*. Table S6. CpGs with p-values <1.0 × 10^-4^ from Epigenome-wide Association Study of DNA Methylation in Child Peripheral Blood with Higher versus Lower Liver Fat Accumulation in Childhood*. [file 13148_2019_799_MOESM4_ESM.docx]

**Table S5 CpGs with p-values <1.0 x 10^-4^ from Epigenome-wide Association Study of DNA Methylation in Cord Blood with Higher versus Lower Liver Fat Accumulation in Childhood^*^**

| CpG | Chromosome | Position | Gene | Effect | SE | P-value |
| --- | --- | --- | --- | --- | --- | --- |
| cg00113096 | 2 | 134119805 | *NCKAP5* | -0.76 | 0.16 | 2.48 x 10^-6^ |
| cg25163851 | 19 | 45996584 | *RTN2* | 2.10 | 0.45 | 3.50 x 10^-6^ |
| cg02252421 | 5 | 127914655 | *FBN2* | -0.87 | 0.19 | 8.32 x 10^-6^ |
| cg17492481 | 5 | 134735068 | *MACROH2A1* | -5.82 | 1.31 | 9.39 x 10^-6^ |
| cg24718356 | 6 | 32822171 | *PSMB9, TAP1* | -5.26 | 1.19 | 9.83 x 10^-6^ |
| cg11937857 | 11 | 36000550 | *LDLRAD3* | -1.11 | 0.25 | 1.11 x 10^-5^ |
| cg02629506 | 22 | 25595814 | *CRYBB3* | -2.10 | 0.49 | 1.53 x 10^-5^ |
| cg05865822 | 10 | 38716268 | *LINC00999* | -1.11 | 0.26 | 2.10 x 10^-5^ |
| cg08287265 | 10 | 101292766 | *NKX2-3* | -2.78 | 0.66 | 2.34 x 10^-5^ |
| cg17104766 | 2 | 66617333 | *AC092669.3* | -0.93 | 0.22 | 2.43 x 10^-5^ |
| cg01182430 | 18 | 75680550 | *LINC01029* | -1.88 | 0.45 | 2.71 x 10^-5^ |
| cg15033034 | 3 | 194106984 | *GP5* | 0.92 | 0.22 | 3.23 x 10^-5^ |
| cg23243857 | 5 | 142001185 | *FGF1* | -1.38 | 0.34 | 3.80 x 10^-5^ |
| cg23042796 | 2 | 89065350 | *ANKRD36BP2* | -3.02 | 0.74 | 4.21 x 10^-5^ |
| cg03795157 | 12 | 133531927 | *ZNF605* | 0.99 | 0.24 | 4.35 x 10^-5^ |
| ch.4.39131871R | 4 | 39455476 | *KLB* | 1.37 | 0.34 | 4.98 x 10^-5^ |
| cg19476186 | 20 | 3869959 | *PANK2* | -5.63 | 1.39 | 4.99 x 10^-5^ |
| cg14750616 | 10 | 24832879 | *KIAA1217* | -2.23 | 0.55 | 5.41 x 10^-5^ |
| cg19697232 | 5 | 16854566 | *MYO10* | -1.02 | 0.25 | 5.54 x 10^-5^ |
| cg20786909 | 8 | 145235410 | *MROH1* | -2.58 | 0.64 | 5.98 x 10^-5^ |
| cg09032137 | 11 | 19362896 | *NAV2* | 1.17 | 0.29 | 6.59 x 10^-5^ |
| cg03925809 | 19 | 6393419 | *GTF2F1* | -3.03 | 0.76 | 6.95 x 10^-5^ |
| cg02073954 | 6 | 28193080 | *ZSCAN9* | -4.15 | 1.05 | 7.81 x 10^-5^ |
| cg25560772 | 17 | 45057470 | *RPRML* | 0.53 | 0.13 | 7.88 x 10^-5^ |
| cg01972651 | 14 | 99613193 | *AL162151.4* | 1.09 | 0.28 | 8.41 x 10^-5^ |
| cg14262490 | 9 | 132630396 | *USP20* | 4.29 | 1.09 | 8.59 x 10^-5^ |
| cg06184262 | 17 | 40610858 | *ATP6V0A1* | 2.63 | 0.67 | 8.77 x 10^-5^ |
| cg26754824 | 7 | 1476361 | *MICALL2* | 2.20 | 0.56 | 9.02 x 10^-5^ |
| cg06399529 | 16 | 4896943 | *UBN1, GLYR1* | 2.99 | 0.77 | 9.69 x 10^-5^ |
| cg25846061 | 2 | 236415673 | *AGAP1* | 1.91 | 0.49 | 9.83 x 10^-5^ |

^*^Effect estimates represent results from logistic regression models for each 10% difference in DNA methylation in newborns. Liver fat was dichotomized into low, ≤2.0%, and high, >2.0%, liver fat accumulation. Associations are adjusted for maternal age, education level, early-pregnancy BMI and smoking, gestational age at birth, child sex, cell type proportions and batch. BMI, Body Mass Index, n, number, SE, standard error.

**Table S6 CpGs with p-values <1.0 x 10^-4^ from Epigenome-wide Association Study of DNA Methylation in Child Peripheral Blood with Higher versus Lower Liver Fat Accumulation in Childhood^*^**

| CpG | Chromosome | Position | Gene | Effect | SE | P-value |
| --- | --- | --- | --- | --- | --- | --- |
| cg26886411 | 13 | 55015275 | *RPL13AP25* | -4.27 | 0.87 | 8.50 x 10^-7^ |
| cg02734482 | 3 | 129761854 | *OR7E21P* | -2.95 | 0.61 | 1.39 x 10^-6^ |
| cg20896197 | 10 | 91461107 | *KIF20B* | -9.50 | 1.97 | 1.50 x 10^-6^ |
| cg07138092 | 8 | 67687406 | *SGK3* | 8.46 | 1.93 | 1.20 x 10^-5^ |
| cg09634659 | 1 | 26678100 | *AIM1L* | 2.08 | 0.48 | 1.28 x 10^-5^ |
| cg18877285 | 11 | 124790815 | *HEPACAM* | -6.35 | 1.46 | 1.42 x 10^-5^ |
| cg16364629 | 3 | 52303212 | *WDR82, MIRLET7G* | 3.74 | 0.87 | 1.71 x 10^-5^ |
| cg03693434 | 17 | 80050880 | *FASN* | -3.04 | 0.71 | 1.74 x 10^-5^ |
| cg16737533 | 18 | 77905355 | *PARD6G-AS1* | -1.05 | 0.24 | 1.83 x 10^-5^ |
| cg07453857 | 3 | 170806983 | *TNIK* | -3.93 | 0.92 | 1.88 x 10^-5^ |
| cg12942606 | 13 | 36429952 | *MIR548F5, DCLK1* | 1.60 | 0.38 | 2.09 x 10^-5^ |
| cg05158615 | 7 | 24323559 | *NPY* | -2.69 | 0.63 | 2.11 x 10^-5^ |
| cg17157462 | 13 | 37268404 | *SERTM1* | 3.19 | 0.76 | 2.63 x 10^-5^ |
| cg18350520 | 17 | 2595812 | *CLUH* | -3.70 | 0.88 | 2.67 x 10^-5^ |
| cg20143970 | 6 | 323570 | *DUSP22* | 3.12 | 0.75 | 3.35 x 10^-5^ |
| cg13155079 | 11 | 44286499 | *ALX4* | -2.86 | 0.69 | 3.36 x 10^-5^ |
| cg12126686 | 19 | 35821634 | *CD22* | 2.36 | 0.57 | 3.38 x 10^-5^ |
| cg03644271 | 11 | 18415989 | *LDHA* | 5.89 | 1.43 | 3.90 x 10^-5^ |
| cg00378658 | 11 | 1232347 | *MUC5AC* | 1.34 | 0.33 | 3.94 x 10^-5^ |
| cg06684201 | 7 | 157447249 | *PTPRN2* | 4.97 | 1.21 | 4.02 x 10^-5^ |
| cg08759899 | 5 | 101831271 | *SLCO6A1* | -3.87 | 0.94 | 4.05 x 10^-5^ |
| cg02167399 | 2 | 239039959 | *ESPNL* | -2.76 | 0.67 | 4.30 x 10^-5^ |
| cg18596636 | 15 | 26915732 | *GABRB3* | -1.27 | 0.31 | 4.39 x 10^-5^ |
| cg02130894 | 6 | 4965241 | *CDYL* | -3.31 | 0.81 | 4.66 x 10^-5^ |
| cg07015412 | 15 | 78025260 | *LINGO1* | -.94 | 0.23 | 4.67 x 10^-5^ |
| cg08372350 | 16 | 88943191 | *CBFA2T3* | -2.96 | 0.73 | 4.91 x 10^-5^ |
| cg24811563 | 5 | 133383131 | *CTB-113I20.2* | -3.19 | 0.79 | 5.05 x 10^-5^ |
| cg19988338 | 13 | 110440179 | *IRS2* | 3.45 | 0.85 | 5.12 x 10^-5^ |
| cg24072283 | 14 | 90798384 | *NRDE2* | 7.79 | 1.93 | 5.61 x 10^-5^ |
| cg24554151 | 11 | 93885823 | *PANX1* | -.95 | 0.24 | 5.77 x 10^-5^ |
| cg03467763 | 2 | 10570059 | *HPCAL1* | -3.32 | 0.83 | 6.03 x 10^-5^ |
| cg13571972 | 9 | 132177348 | *RP11-65J3.3* | -1.23 | 0.31 | 6.77 x 10^-5^ |
| cg19196182 | 12 | 99038046 | *IKBIP, APAF1* | -4.76 | 1.20 | 7.18 x 10^-5^ |
| cg25470324 | 2 | 43546568 | *THADA* | 2.72 | 0.69 | 7.52 x 10^-5^ |
| cg00363811 | 10 | 103113695 | *BTRC* | -4.05 | 1.03 | 7.80 x 10^-5^ |
| cg12878228 | 7 | 142456124 | *PRSS1* | -2.63 | 0.67 | 7.88 x 10^-5^ |
| cg06878009 | 16 | 77874011 | *VAT1L* | 1.94 | 0.49 | 8.20 x 10^-5^ |
| cg12000458 | 17 | 19192696 | *EPN2* | -3.73 | 0.95 | 8.27 x 10^-5^ |
| cg07705639 | 1 | 174992861 | *MRPS14* | 1.42 | 0.36 | 8.41 x 10^-5^ |
| cg06391982 | 3 | 13936753 | *WNT7A* | 1.68 | 0.43 | 8.59 x 10^-5^ |
| cg25935716 | 20 | 30102579 | *HM13* | -10.69 | 2.73 | 8.97 x 10^-5^ |
| cg26579556 | 14 | 103673450 | *RP11-736N17.9* | .87 | 0.22 | 9.27 x 10^-5^ |
| cg13260976 | 10 | 111727104 | *ADD3-AS1* | 1.67 | 0.43 | 9.48 x 10^-5^ |
| cg08737743 | 6 | 123318023 | *CLVS2* | -1.39 | 0.36 | 9.73 x 10^-5^ |
| cg23418198 | 17 | 4925746 | *KIF1C* | 4.95 | 1.27 | 9.84 x 10^-5^ |
| cg23662448 | 12 | 109797184 | *LINC01486* | -3.21 | 0.82 | 9.98 x 10^-5^ |

^*^Effect estimates represent results from logistic regression models for each 10% difference in DNA methylation in 10-year-old children. Liver fat was dichotomized into low, ≤2.0%, and high, >2.0%, liver fat accumulation. Associations are adjusted for maternal age, education level, early-pregnancy BMI and smoking, child age at measurement, child sex, cell type proportions and batch. BMI, Body Mass Index, n, number, SE, standard error.
