# Supplementary material for: Newborn and childhood differential DNA methylation and liver fat in school-age children
Source: Clin Epigenetics. 2019 Dec 31;12:3. doi: 10.1186/s13148-019-0799-6 (PMC6938624; doi:10.1186/s13148-019-0799-6)
Supplement: Supplementary file 5 — Additional file 5: Table S7. Results of Basic Model and Child BMI Model for CpGs with p-values <1.0 × 10^-4^ in Main Model in Cord Blood*. Table S8. Results of Basic Model and Child BMI Model for CpGs with p-values <1.0 × 10^-4^ in Main Model in Child Peripheral Blood*. [file 13148_2019_799_MOESM5_ESM.docx]

**Table S7 Results of Basic Model and Child BMI Model for CpGs with p-values <1.0 x 10^-4^ in Main Model in Cord Blood^*^**

|  | **Main model** | | | **Basic Model** | | | **Child BMI model** | | |
| --- | --- | --- | --- | --- | --- | --- | --- | --- | --- |
| CpG | Effect | SE | P-value | Effect | SE | P-value | Effect | SE | P-value |
| cg05527491 | 0.39 | 0.08 | 3.31 x 10^-6^ | 0.38 | 0.08 | 3.24 x 10^-6^ | 0.34 | 0.08 | 2.37 x 10^-5^ |
| cg16530382 | 0.05 | 0.01 | 5.59 x 10^-6^ | 0.05 | 0.01 | 4.75 x 10^-6^ | 0.05 | 0.11 | 3.25 x 10^-6^ |
| cg08287265 | -0.34 | 0.08 | 8.94 x 10^-6^ | -0.32 | 0.08 | 3.03 x 10^-5^ | -0.31 | 0.08 | 6.79 x 10^-5^ |
| cg24718356 | -0.65 | 0.15 | 9.41 x 10^-6^ | -0.65 | 0.15 | 9.40 x 10^-6^ | -0.64 | 0.14 | 5.46 x 10^-6^ |
| cg08570275 | 0.34 | 0.08 | 9.65 x 10^-6^ | 0.30 | 0.07 | 6.73 x 10^-5^ | 0.27 | 0.07 | 1.15 x 10^-4^ |
| cg01396580 | 0.81 | 0.18 | 1.19 x 10^-5^ | 0.80 | 0.18 | 1.22 x 10^-5^ | 0.70 | 0.18 | 9.64 x 10^-5^ |
| cg12704462 | 0.28 | 0.06 | 1.58 x 10^-5^ | 0.27 | 0.06 | 3.74 x 10^-5^ | 0.24 | 0.06 | 3.76 x 10^-5^ |
| cg15033034 | 0.11 | 0.03 | 1.66 x 10^-5^ | 0.11 | 0.03 | 2.33 x 10^-5^ | 0.10 | 0.02 | 1.78 x 10^-5^ |
| cg18841298 | -0.09 | 0.02 | 1.70 x 10^-5^ | -0.09 | 0.02 | 7.93 x 10^-6^ | -0.08 | 0.02 | 6.20 x 10^-5^ |
| cg13987088 | 0.16 | 0.04 | 1.72 x 10^-5^ | 0.16 | 0.04 | 2.42 x 10^-5^ | 0.13 | 0.04 | 5.67 x 10^-4^ |
| cg13223721 | -0.21 | 0.05 | 1.83 x 10^-5^ | -0.22 | 0.05 | 9.85 x 10^-6^ | -0.19 | 0.05 | 6.99 x 10^-5^ |
| cg05551922 | -0.20 | 0.05 | 1.92 x 10^-5^ | -0.20 | 0.05 | 2.73 x 10^-5^ | -0.18 | 0.05 | 7.70 x 10^-5^ |
| cg00113096 | -0.09 | 0.02 | 2.04 x 10^-5^ | -0.07 | 0.02 | 6.83 x 10^-5^ | -0.06 | 0.02 | 1.38 x 10^-3^ |
| cg10368518 | -0.48 | 0.11 | 2.23 x 10^-5^ | -0.48 | 0.11 | 2.15 x 10^-5^ | -0.46 | 0.12 | 1.54 x 10^-4^ |
| cg16530177 | -0.09 | 0.02 | 2.75 x 10^-5^ | -0.09 | 0.02 | 2.69 x 10^-5^ | -0.08 | 0.02 | 1.58 x 10^-5^ |
| cg23915314 | -0.50 | 0.12 | 2.99 x 10^-5^ | -0.50 | 0.12 | 2.91 x 10^-5^ | -0.50 | 0.11 | 1.18 x 10^-5^ |
| cg17492481 | -0.67 | 0.16 | 3.25 x 10^-5^ | -0.68 | 0.16 | 3.18 x 10^-5^ | -0.59 | 0.17 | 6.45 x 10^-4^ |
| cg12732390 | -0.10 | 0.02 | 4.66 x 10^-5^ | -0.08 | 0.02 | 4.03 x 10^-4^ | -0.07 | 0.02 | 6.29 x 10^-4^ |
| cg24673605 | 0.13 | 0.03 | 4.69 x 10^-5^ | 0.13 | 0.03 | 5.63 x 10^-5^ | 0.12 | 0.03 | 5.21 x 10^-5^ |
| cg09274253 | 0.22 | 0.06 | 4.78 x 10^-5^ | 0.20 | 0.05 | 2.15 x 10^-4^ | 0.18 | 0.05 | 2.30 x 10^-4^ |
| cg08882503 | 0.21 | 0.05 | 5.16 x 10^-5^ | 0.19 | 0.05 | 5.11 x 10^-4^ | 0.18 | 0.05 | 1.88 x 10^-3^ |
| cg18554922 | 0.22 | 0.05 | 5.31 x 10^-5^ | 0.22 | 0.05 | 7.41 x 10^-5^ | 0.18 | 0.05 | 3.65 x 10^-5^ |
| cg27065979 | -0.27 | 0.07 | 5.37 x 10^-5^ | -0.28 | 0.07 | 2.33 x 10^-5^ | -0.27 | 0.06 | 2.88 x 10^-5^ |
| cg19817544 | -0.16 | 0.04 | 5.58 x 10^-5^ | -0.16 | 0.04 | 4.86 x 10^-5^ | -0.16 | 0.04 | 5.40 x 10^-5^ |
| cg25751266 | -0.45 | 0.11 | 5.86 x 10^-5^ | -0.45 | 0.11 | 5.61 x 10^-5^ | -0.47 | 0.10 | 7.22 x 10^-6^ |
| cg02397368 | -0.12 | 0.03 | 6.34 x 10^-5^ | -0.12 | 0.03 | 6.09 x 10^-5^ | -0.12 | 0.03 | 3.96 x 10^-5^ |
| cg23042796 | -0.33 | 0.08 | 6.91 x 10^-5^ | -0.33 | 0.08 | 6.45 x 10^-5^ | -0.29 | 0.08 | 3.83 x 10^-4^ |
| cg16315928 | -0.56 | 0.14 | 7.35 x 10^-5^ | -0.56 | 0.14 | 7.03 x 10^-5^ | -0.48 | 0.14 | 4.97 x 10^-4^ |
| cg21847720 | 0.02 | 0.01 | 8.10 x 10^-5^ | 0.02 | 0.01 | 7.89 x 10^-5^ | 0.02 | 0.01 | 8.28 x 10^-5^ |
| cg02063915 | 0.35 | 0.09 | 8.25 x 10^-5^ | 0.34 | 0.09 | 1.36 x 10^-4^ | 0.29 | 0.08 | 2.62 x 10^-4^ |
| cg16052901 | 0.11 | 0.03 | 8.42 x 10^-5^ | 0.11 | 0.03 | 9.01 x 10^-5^ | 0.09 | 0.03 | 3.39 x 10^-4^ |
| cg18296281 | 0.99 | 0.25 | 9.04 x 10^-5^ | 0.99 | 0.25 | 9.13 x 10^-5^ | 0.88 | 0.25 | 4.27 x 10^-4^ |

^*^Effect estimates represent the change in liver fat fraction (%) per 10% difference in DNA methylation beta and standard error in newborns. The main model was adjusted for: maternal age, education level, early-pregnancy BMI and smoking, gestational age at birth, child sex, cell type proportions and batch. The basic model was adjusted for: gestational age at birth, child sex, cell type proportions and batch. The childhood BMI model was adjusted for: main model additionally adjusted for childhood BMI at 10 years. BMI, Body Mass Index, n, number, SE, standard error.

# **Table S8 Results of Basic Model and Child BMI Model for CpGs with p-values <1.0 x 10^-4^ in Main Model in Child Peripheral Blood^*^**

|  | **Main model** | | | **Basic Model** | | | **Child BMI model** | | |
| --- | --- | --- | --- | --- | --- | --- | --- | --- | --- |
| CpG | Effect | SE | P-value | Effect | SE | P-value | Effect | SE | P-value |
| cg12020444 | 0.30 | 0.06 | 1.54 x 10^-7^ | 0.28 | 0.06 | 7.96 x 10^-6^ | 0.30 | 0.06 | 7.61 x 10^-7^ |
| cg00946960 | 0.15 | 0.03 | 1.44 x 10^-6^ | 0.17 | 0.03 | 5.67 x 10^-7^ | 0.15 | 0.03 | 3.83 x 10^-6^ |
| cg10573751 | -0.16 | 0.03 | 1.59 x 10^-6^ | -0.19 | 0.04 | 8.33 x 10^-8^ | -0.16 | 0.04 | 3.75 x 10^-5^ |
| cg19497388 | -0.41 | 0.09 | 4.96 x 10^-6^ | -0.43 | 0.10 | 2.62 x 10^-5^ | -0.43 | 0.09 | 2.41 x 10^-6^ |
| cg00549910 | -0.74 | 0.16 | 5.78 x 10^-6^ | -0.77 | 0.18 | 2.07 x 10^-5^ | -0.73 | 0.17 | 1.54 x 10^-5^ |
| cg13571972 | -0.15 | 0.03 | 6.91 x 10^-6^ | -0.15 | 0.03 | 1.60 x 10^-6^ | -0.15 | 0.03 | 3.49 x 10^-6^ |
| cg05837235 | 0.69 | 0.15 | 7.70 x 10^-6^ | 0.65 | 0.16 | 6.77 x 10^-6^ | 0.70 | 0.17 | 3.74 x 10^-5^ |
| cg16555595 | 0.45 | 0.11 | 7.75 x 10^-6^ | 0.44 | 0.11 | 1.17 x 10^-4^ | 0.43 | 0.10 | 2.70 x 10^-5^ |
| cg17051207 | 0.22 | 0.05 | 8.26 x 10^-6^ | 0.22 | 0.05 | 2.43 x 10^-5^ | 0.21 | 0.05 | 2.06 x 10^-5^ |
| cg00378658 | 0.15 | 0.03 | 8.35 x 10^-6^ | 0.15 | 0.04 | 6.30 x 10^-5^ | 0.15 | 0.03 | 1.60 x 10^-6^ |
| cg01871127 | 0.40 | 0.09 | 8.47 x 10^-6^ | 0.46 | 0.09 | 4.86 x 10^-7^ | 0.38 | 0.09 | 6.06 x 10^-5^ |
| cg01282508 | -0.04 | 0.01 | 8.85 x 10^-6^ | -0.04 | 0.01 | 9.99 x 10^-5^ | -0.03 | 0.01 | 6.86 x 10^-5^ |
| cg23763836 | 0.73 | 0.17 | 9.70 x 10^-6^ | 0.62 | 0.19 | 8.05 x 10^-4^ | 0.71 | 0.16 | 1.60 x 10^-5^ |
| cg19398365 | -0.39 | 0.09 | 1.01 x 10^-5^ | -0.37 | 0.09 | 5.49 x 10^-5^ | -0.34 | 0.09 | 2.38 x 10^-4^ |
| cg18471160 | -0.54 | 0.12 | 1.18 x 10^-5^ | -0.59 | 0.13 | 1.37 x 10^-5^ | -0.50 | 0.12 | 2.34 x 10^-5^ |
| cg20489847 | -0.42 | 0.10 | 1.19 x 10^-5^ | -0.44 | 0.11 | 2.97 x 10^-5^ | -0.42 | 0.10 | 0.38 x 10^-5^ |
| cg18857369 | -0.31 | 0.07 | 1.21 x 10^-5^ | -0.31 | 0.07 | 2.57 x 10^-6^ | -0.35 | 0.07 | 1.00 x 10^-6^ |
| cg05511958 | -0.32 | 0.08 | 1.35 x 10^-5^ | -0.30 | 0.08 | 5.80 x 10^-5^ | -0.31 | 0.08 | 3.64 x 10^-5^ |
| cg09071239 | -0.35 | 0.08 | 1.36 x 10^-5^ | -0.34 | 0.09 | 3.06 x 10^-4^ | -0.34 | 0.08 | 5.08 x 10^-5^ |
| cg00167491 | -0.46 | 0.11 | 1.86 x 10^-5^ | -0.53 | 0.11 | 2.54 x 10^-6^ | -0.46 | 0.11 | 1.78 x 10^-5^ |
| cg02110701 | -0.83 | 0.19 | 1.87 x 10^-5^ | -0.88 | 0.24 | 2.05 x 10^-4^ | -0.87 | 0.21 | 4.63 x 10^-5^ |
| cg13680337 | 0.15 | 0.04 | 1.97 x 10^-5^ | 0.15 | 0.04 | 7.50 x 10^-5^ | 0.16 | 0.03 | 1.43 x 10^-6^ |
| cg20896197 | -0.82 | 0.19 | 2.14 x 10^-5^ | -0.71 | 0.21 | 8.68 x 10^-4^ | -0.73 | 0.20 | 2.16 x 10^-4^ |
| cg26579556 | 0.12 | 0.03 | 2.18 x 10^-5^ | 0.11 | 0.03 | 7.94 x 10^-5^ | 0.10 | 0.03 | 2.82 x 10^-4^ |
| cg26886411 | -0.40 | 0.09 | 2.19 x 10^-5^ | -0.38 | 0.10 | 1.55 x 10^-4^ | -0.37 | 0.10 | 1.91 x 10^-4^ |
| cg08264376 | -0.23 | 0.05 | 2.28 x 10^-5^ | -0.24 | 0.05 | 8.27 x 10^-6^ | -0.21 | 0.05 | 1.11 x 10^-4^ |
| cg23303505 | -0.06 | 0.01 | 2.40 x 10^-5^ | -0.06 | 0.01 | 4.95 x 10^-6^ | -0.06 | 0.01 | 1.11 x 10^-4^ |
| cg01294686 | -0.33 | 0.08 | 241 x 10^-5^ | -0.30 | 0.09 | 5.64 x 10^-4^ | -0.29 | 0.08 | 3.48 x 10^-4^ |
| cg13155079 | -0.34 | 0.08 | 2.43 x 10^-5^ | -0.33 | 0.09 | 1.54 x 10^-4^ | -0.34 | 0.09 | 1.79 x 10^-4^ |
| cg24407942 | -0.50 | 0.12 | 2.45 x 10^-5^ | -0.48 | 0.12 | 1.22 x 10^-4^ | -0.51 | 0.11 | 5.99 x 10^-6^ |
| cg03290827 | 0.22 | 0.05 | 2.48 x 10^-5^ | 0.22 | 0.06 | 1.25 x 10^-5^ | 0.22 | 0.05 | 1.95 x 10^-5^ |
| cg06769094 | 0.67 | 0.16 | 2.49 x 10^-5^ | 0.66 | 0.17 | 7.36 x 10^-5^ | 0.68 | 0.17 | 3.89 x 10^-5^ |
| cg15594605 | 0.04 | 0.01 | 2.54 x 10^-5^ | 0.04 | 0.01 | 8.19 x 10^-5^ | 0.04 | 0.01 | 5.36 x 10^-5^ |
| cg27107150 | -0.30 | 0.07 | 2.57 x 10^-5^ | -0.31 | 0.07 | 6.82 x 10^-6^ | -0.31 | 0.07 | 8.63 x 10^-6^ |
| cg12000458 | -0.41 | 0.10 | 2.63 x 10^-5^ | -0.41 | 0.11 | 2.61 x 10^-4^ | -0.42 | 0.09 | 5.47 x 10^-6^ |
| cg23055496 | 0.20 | 0.05 | 2.63 x 10^-5^ | 0.21 | 0.05 | 4.32 x 10^-5^ | 0.19 | 0.05 | 2.66 x 10^-4^ |
| cg16244155 | -0.10 | 0.01 | 2.97 x 10^-5^ | -0.10 | 0.01 | 1.91 x 10^-4^ | -0.09 | 0.01 | 1.34 x 10^-5^ |
| cg19310458 | 0.25 | 0.06 | 3.04 x 10^-5^ | 0.22 | 0.07 | 7.85 x 10^-4^ | 0.24 | 0.06 | 1.38 x 10^-4^ |
| cg12061285 | -0.44 | 0.11 | 3.31 x 10^-5^ | -0.40 | 0.11 | 4.95 x 10^-4^ | -0.42 | 0.11 | 1.01 x 10^-4^ |
| cg05251669 | -0.09 | 0.01 | 3.33 x 10^-5^ | -0.09 | 0.01 | 1.29 x 10^-4^ | -0.09 | 0.01 | 6.50 x 10^-5^ |
| cg14694712 | 0.16 | 0.04 | 3.53 x 10^-5^ | 0.19 | 0.04 | 3.34 x 10^-6^ | 0.16 | 0.04 | 2.23 x 10^-4^ |
| cg10829004 | -0.38 | 0.09 | 3.83 x 10^-5^ | -0.37 | 0.11 | 5.44 x 10^-4^ | -0.35 | 0.09 | 1.67 x 10^-4^ |
| cg04139359 | -0.30 | 0.07 | 3.89 x 10^-5^ | -0.29 | 0.09 | 7.84 x 10^-4^ | -0.27 | 0.08 | 6.30 x 10^-4^ |
| cg17515773 | -0.46 | 0.11 | 4.11 x 10^-5^ | -0.46 | 0.13 | 3.68 x 10^-4^ | -0.44 | 0.11 | 9.78 x 10^-5^ |
| cg25213720 | 0.23 | 0.06 | 4.11 x 10^-5^ | 0.25 | 0.06 | 1.60 x 10^-5^ | 0.25 | 0.06 | 1.90 x 10^-5^ |
| cg17382048 | 0.20 | 0.05 | 4.20 x 10^-5^ | 0.21 | 0.06 | 1.43 x 10^-4^ | 0.17 | 0.05 | 4.40 x 10^-4^ |
| cg25084220 | 1.59 | 0.39 | 4.28 x 10^-5^ | 1.69 | 0.41 | 4.65 x 10^-5^ | 1.57 | 0.41 | 1.27 x 10^-4^ |
| cg01817897 | -0.34 | 0.08 | 4.52 x 10^-5^ | -0.36 | 0.09 | 3.24 x 10^-5^ | -0.30 | 0.09 | 4.90 x 10^-4^ |
| cg09321758 | 0.47 | 0.12 | 4.74 x 10^-5^ | 0.43 | 0.14 | 1.72 x 10^-3^ | 0.46 | 0.12 | 8.76 x 10^-5^ |
| cg21405786 | -0.16 | 0.04 | 4.84 x 10^-5^ | -0.17 | 0.04 | 2.57 x 10^-5^ | -0.14 | 0.04 | 8.00 x 10^-4^ |
| cg14040899 | -0.36 | 0.09 | 5.08 x 10^-5^ | -0.40 | 0.09 | 4.51 x 10^-6^ | -0.34 | 0.09 | 2.97 x 10^-4^ |
| cg01031312 | -0.19 | 0.05 | 5.10 x 10^-5^ | -0.18 | 0.06 | 2.10 x 10^-3^ | -0.21 | 0.05 | 3.80 x 10^-5^ |
| cg07015412 | -0.09 | 0.01 | 5.17 x 10^-5^ | -0.09 | 0.01 | 2.64 x 10^-4^ | -0.09 | 0.01 | 8.92 x 10^-5^ |
| cg26216343 | -0.50 | 0.12 | 5.18 x 10^-5^ | -0.50 | 0.13 | 7.92 x 10^-5^ | -0.45 | 0.12 | 1.82 x 10^-4^ |
| cg17235827 | 0.40 | 0.10 | 5.55 x 10^-5^ | 0.48 | 0.10 | 3.24 x 10^-6^ | 0.40 | 0.11 | 4.11 x 10^-4^ |
| cg18994015 | 0.14 | 0.03 | 6.30 x 10^-5^ | 0.15 | 0.04 | 1.08 x 10^-4^ | 0.13 | 0.04 | 2.65 x 10^-4^ |
| cg24957532 | -0.24 | 0.06 | 6.42 x 10^-5^ | -0.20 | 0.06 | 1.14 x 10^-3^ | -0.20 | 0.06 | 7.24 x 10^-4^ |
| cg04496920 | 0.15 | 0.04 | 6.66 x 10^-5^ | 0.15 | 0.04 | 8.31 x 10^-4^ | 0.15 | 0.04 | 3.37 x 10^-4^ |
| cg13657092 | 0.46 | 0.12 | 6.70 x 10^-5^ | 0.43 | 0.13 | 1.04 x 10^-3^ | 0.49 | 0.11 | 1.34 x 10^-5^ |
| cg19430975 | 0.67 | 0.17 | 6.93 x 10^-5^ | 0.62 | 0.17 | 3.21 x 10^-4^ | 0.69 | 0.15 | 6.12 x 10^-6^ |
| cg16737533 | -0.11 | 0.03 | 6.96 x 10^-5^ | -0.11 | 0.03 | 8.19 x 10^-5^ | -0.10 | 0.03 | 1.20 x 10^-4^ |
| cg02734482 | -0.26 | 0.06 | 7.06 x 10^-5^ | -0.27 | 0.06 | 2.13 x 10^-5^ | -0.25 | 0.06 | 9.71 x 10^-5^ |
| cg12128483 | 0.15 | 0.04 | 7.16 x 10^-5^ | 0.14 | 0.04 | 1.52 x 10^-3^ | 0.15 | 0.04 | 2.75 x 10^-4^ |
| cg23559165 | 0.26 | 0.07 | 7.18 x 10^-5^ | 0.26 | 0.07 | 3.83 x 10^-4^ | 0.26 | 0.08 | 7.15 x 10^-4^ |
| cg05352250 | -0.39 | 0.10 | 7.22 x 10^-5^ | -0.40 | 0.10 | 8.83 x 10^-5^ | -0.38 | 0.11 | 3.34 x 10^-4^ |
| cg13071609 | -0.31 | 0.08 | 7.28 x 10^-5^ | -0.32 | 0.08 | 7.18 x 10^-5^ | -0.29 | 0.08 | 2.76 x 10^-4^ |
| cg16991637 | -0.16 | 0.04 | 7.48 x 10^-5^ | -0.17 | 0.04 | 1.19 x 10^-4^ | -0.13 | 0.04 | 4.28 x 10^-3^ |
| cg04815301 | 0.37 | 0.09 | 7.76 x 10^-5^ | 0.37 | 0.10 | 1.00 x 10^-3^ | 0.33 | 0.10 | 5.19 x 10^-4^ |
| cg13985132 | 0.11 | 0.03 | 7.77 x 10^-5^ | 0.13 | 0.03 | 2.45 x 10^-5^ | 0.10 | 0.03 | 4.67 x 10^-4^ |
| cg08759899 | -0.38 | 0.10 | 7.91 x 10^-5^ | -0.43 | 0.10 | 2.36 x 10^-5^ | -0.32 | 0.10 | 8.53 x 10^-4^ |
| cg03693434 | -0.33 | 0.08 | 8.20 x 10^-5^ | -0.37 | 0.09 | 2.09 x 10^-5^ | -0.31 | 0.08 | 2.56 x 10^-4^ |
| cg21553199 | 0.22 | 0.05 | 8.54 x 10^-5^ | 0.21 | 0.06 | 2.26 x 10^-4^ | 0.19 | 0.05 | 2.84 x 10^-4^ |
| cg01953119 | -0.47 | 0.19 | 8.72 x 10^-5^ | -0.48 | 0.13 | 1.22 x 10^-4^ | -0.44 | 0.12 | 1.97 x 10^-4^ |
| cg14315558 | -0.46 | 0.12 | 9.37 x 10^-5^ | -0.51 | 0.13 | 6.52 x 10^-5^ | -0.45 | 0.12 | 1.17 x 10^-4^ |
| cg27033805 | -0.09 | 0.01 | 9.59 x 10^-5^ | -0.10 | 0.01 | 1.97 x 10^-4^ | -0.08 | -0.01 | 6.11 x 10^-4^ |
| cg10177518 | 0.08 | 0.01 | 9.86 x 10^-5^ | 0.09 | 0.01 | 6.48 x 10^-5^ | 0.08 | 0.01 | 3.11 x 10^-4^ |

^*^Effect estimates represent the change in liver fat fraction (%) per 10% difference in DNA methylation beta and standard error in newborns. The main model was adjusted for: maternal age, education level, early-pregnancy BMI and smoking, child age at measurement, child sex, cell type proportions and batch. The basic model was adjusted for: child age at measurement, child sex, cell type proportions and batch. The childhood BMI model was adjusted for: main model additionally adjusted for childhood BMI at 10 years. BMI, Body Mass Index, n, number, SE, standard error.
