# Supplementary material for: Newborn and childhood differential DNA methylation and liver fat in school-age children
Source: Clin Epigenetics. 2019 Dec 31;12:3. doi: 10.1186/s13148-019-0799-6 (PMC6938624; doi:10.1186/s13148-019-0799-6)
Supplement: Supplementary file 6 — Additional file 6: Table S9. Significantly enriched GO terms among CpGs with p-values <1.0 × 10^-4^ Identified in Cord Blood Associated with Liver Fat Accumulation in Childhood. Table S10. Significantly enriched GO terms among CpGs with p-values <1.0 × 10^-4^ Identified in Child Peripheral Blood Associated with Liver Fat Accumulation in Childhood. [file 13148_2019_799_MOESM6_ESM.docx]

**Table S9 Significantly enriched GO terms among CpGs with p-values <1.0 x 10^-4^ Identified in Cord Blood Associated with Liver Fat Accumulation in Childhood**

| Category | Term | Count | % | Genes | Fold enrichment | Fisher Exact |
| --- | --- | --- | --- | --- | --- | --- |
| GOTERM_MF_FAT | GO:0019904~Protein domain specific binding | 6 | 19.4 | *CIT, EHMT1, GPX1, MICAL1, NRL, ROBO1* | 6.6 | 2.2 x 10^-4^ |
| GOTERM_BP_FAT | GO:2000116~Regulation of cysteine-type endopeptidase activity | 4 | 12.9 | *GPX1, MICAL1, PSMB9, ROBO1* | 11.9 | 3.2 x 10^-4^ |
| GOTERM_MF_FAT | GO:0030275~LRR domain binding | 2 | 6.5 | *NRL, ROBO1* | 74.8 | 3.2 x 10^-4^ |
| GOTERM_BP_FAT | GO:0033599~Regulation of mammary gland epithelial cell proliferation | 2 | 6.5 | *GPX1, ROBO1* | 74.0 | 3.3 x 10^-4^ |
| GOTERM_BP_FAT | GO:0006641~Triglyceride metabolic process | 3 | 9.7 | *NKX2-3, GPX1, LMF1* | 19.4 | 4.8 x 10^-4^ |
| GOTERM_MF_FAT | GO:0017016~Ras GTPase binding | 4 | 12.9 | *DENND5A, VPS52, CIT, MICAL1* | 10.0 | 6.1 x 10^-4^ |
| GOTERM_MF_FAT | GO:0017124~SH3 domain binding | 3 | 9.7 | *CIT, GPX1, MICAL1* | 17.0 | 7.0 x 10^-4^ |
| GOTERM_BP_FAT | GO:0006639~Acylglycerol metabolic process | 3 | 9.7 | *NKX2-3, GPX1, LMF1* | 16.9 | 7.1 x 10^-4^ |
| GOTERM_BP_FAT | GO:0006638~Neutral lipid metabolic process | 3 | 9.7 | *NKX2-3, GPX1, LMF1* | 16.8 | 7.3 x 10^-4^ |
| GOTERM_MF_FAT | GO:0031267~small GTPase binding | 4 | 12.9 | *DENND5A, VPS52, CIT, MICAL1* | 9.3 | 8.2 x 10^-4^ |
| GOTERM_MF_FAT | GO:0017137~Rab GTPase binding | 3 | 9.7 | *DENND5A, VPS52, MICAL1* | 15.1 | 9.9 x 10^-4^ |
| GOTERM_BP_FAT | GO:0048565~Digestive tract development | 3 | 9.7 | *NKX2-3, VPS52, ACVR2B* | 15.0 | 1.0 x 10^-3^ |
| GOTERM_MF_FAT | GO:0043531~ADP binding | 2 | 6.5 | *P2RY1, TAP1* | 40.8 | 1.1 x 10^-3^ |
| GOTERM_MF_FAT | GO:0051020~GTPase binding | 4 | 12.9 | *DENND5A, VPS52, CIT, MICAL1* | 8.5 | 1.1 x 10^-3^ |
| GOTERM_BP_FAT | GO:0055123~Digestive system development | 3 | 9.7 | *NKX2-3, VPS52, ACVR2B* | 13.8 | 1.3 x 10^-3^ |
| GOTERM_CC_FAT | GO:0031672~A band | 2 | 6.5 | *MYOM2, MYL3* | 33.2 | 1.6 x 10^-3^ |
| GOTERM_MF_FAT | GO:0008307~Structural constituent of muscle | 2 | 6.5 | *MYOM2, MYL3* | 32.0 | 1.8 x 10^-3^ |
| GOTERM_BP_FAT | GO:0042461~Photoreceptor cell development | 2 | 6.5 | *NPHP4, NRL* | 31.7 | 1.8 x 10^-3^ |
| GOTERM_BP_FAT | GO:0052548~Regulation of endopeptidase activity | 4 | 12.9 | *GPX1, MICAL1, PSMB9, ROBO1* | 7.2 | 2.1 x 10^-3^ |
| GOTERM_MF_FAT | GO:0097110~Scaffold protein binding | 2 | 6.5 | *CIT, P2RY1* | 28.0 | 2.3 x 10^-3^ |
| GOTERM_BP_FAT | GO:0048546~Digestive tract morphogenesis | 2 | 6.5 | *NKX2-3, ACVR2B* | 26.6 | 2.5 x 10^-3^ |
| GOTERM_BP_FAT | GO:0052547~Regulation of peptidase activity | 4 | 12.9 | *GPX1, MICAL1, PSMB9, ROBO1* | 6.7 | 2.7 x 10^-3^ |
| GOTERM_BP_FAT | GO:0051336~Regulation of hydrolase activity | 7 | 22.6 | *DENND5A, GPX1, LMF1, MICAL1, MYL3, PSMB9, ROBO1* | 3.5 | 2.8 x 10^-3^ |
| GOTERM_BP_FAT | GO:0043281~Regulation of cysteine-type endopeptidase activity involved in apoptotic process | 3 | 9.7 | *GPX1, MICAL1, ROBO1* | 10.0 | 3.2 x 10^-3^ |
| GOTERM_BP_FAT | GO:0046530~Photoreceptor cell differentiation | 2 | 6.5 | *NPHP4, NRL* | 23.4 | 3.3 x 10^-3^ |
| GOTERM_BP_FAT | GO:0022008~Neurogenesis | 7 | 22.6 | *NEK3, CIT, GP5, NPHP4, NRL, P2RY1, ROBO1* | 3.2 | 4.4 x 10^-3^ |
| GOTERM_BP_FAT | GO:0043534~Blood vessel endothelial cell migration | 2 | 6.5 | *GPX1, ROBO1* | 19.3 | 4.8 x 10^-3^ |
| GOTERM_BP_FAT | GO:0042060~Wound healing | 4 | 12.9 | *F13A1, GPX1, GP5, P2RY1* | 4.9 | 8.2 x 10^-3^ |
| GOTERM_CC_FAT | GO:0098791~Golgi subcompartment | 3 | 9.7 | *DENND5A, VPS52, CIT* | 6.3 | 1.2 x 10^-2^ |
| GOTERM_BP_FAT | GO:0048666~Neuron development | 5 | 16.1 | *NEK3, GP5, NPHP4, NRL, ROBO1* | 3.5 | 1.3 x 10^-2^ |
| GOTERM_BP_FAT | GO:0032940~Secretion by cell | 5 | 16.1 | *VPS52, ACVR2B, F13A1, LMF1, P2RY1* | 3.4 | 1.3 x 10^-2^ |
| GOTERM_BP_FAT | GO:0001654~Eye development | 3 | 9.7 | *ACVR2B, NPHP4, NRL* | 6.0 | 1.3 x 10^-2^ |
| GOTERM_BP_FAT | GO:0048699~Generation of neurons | 6 | 19.4 | *NEK3, CIT, GP5, NPHP4, NRL, ROBO1* | 2.9 | 1.3 x 10^-2^ |
| GOTERM_BP_FAT | GO:0007596~Blood coagulation | 3 | 9.7 | *F13A1, GP5, P2RY1* | 5.8 | 1.4 x 10^-2^ |
| GOTERM_CC_FAT | GO:0031984~organelle subcompartment | 3 | 9.7 | *DENND5A, VPS52, CIT* | 5.9 | 1.4 x 10^-2^ |
| GOTERM_BP_FAT | GO:0009887~Organ morphogenesis | 5 | 16.1 | *NKX2-3, ACVR2B, MYL3, NRL, PSMB9* | 3.4 | 1.5 x 10^-2^ |
| GOTERM_BP_FAT | GO:0009611~Response to wounding | 4 | 12.9 | *F13A1, GPX1, GP5, P2RY1* | 4.1 | 1.5 x 10^-2^ |
| GOTERM_BP_FAT | GO:0006464~Cellular protein modification process | 11 | 35.5 | *NEK3, B3GNT9, ACVR2B, CIT, F13A1, EHMT1, GPX1, LMF1, MICAL1, PSMB9, P2RY1* | 1.9 | 1.6 x 10^-2^ |
| GOTERM_BP_FAT | GO:0036211~Protein modification process | 11 | 35.5 | *NEK3, B3GNT9, ACVR2B, CIT, F13A1, EHMT1, GPX1, LMF1, MICAL1, PSMB9, P2RY1* | 1.9 | 1.6 x 10^-2^ |
| GOTERM_BP_FAT | GO:0007599~Hemostasis | 3 | 9.7 | *F13A1, GP5, P2RY1* | 5.6 | 1.6 x 10^-2^ |
| GOTERM_BP_FAT | GO:0050817~Coagulation | 3 | 9.7 | *F13A1, GP5, P2RY1* | 5.6 | 1.6 x 10^-2^ |
| GOTERM_BP_FAT | GO:0030029~Actin filament-based process | 4 | 12.9 | *CIT, MICAL1, MYL3, NPHP4* | 4.0 | 1.7 x 10^-2^ |
| GOTERM_BP_FAT | GO:0050790~Regulation of catalytic activity | 8 | 25.8 | *DENND5A, ACVR2B, GPX1, LMF1, MICAL1, MYL3, PSMB9, ROBO1* | 2.3 | 1.8 x 10^-2^ |
| GOTERM_BP_FAT | GO:0065009~Regulation of molecular function | 9 | 29.0 | *DENND5A, ACVR2B, GPX1, LMF1, MICAL1, MYL3, PSMB9, P2RY1, ROBO1* | 2.1 | 1.9 x 10^-2^ |
| GOTERM_MF_FAT | GO:0005524~ATP binding | 6 | 19.4 | *NEK3, ACVR2B, CIT, DCK, P2RY1, TAP1* | 2.7 | 1.9 x 10^-2^ |
| GOTERM_BP_FAT | GO:0030162~Regulation of proteolysis | 4 | 12.9 | *GPX1, MICAL1, PSMB9, ROBO1* | 3.8 | 2.0 x 10^-2^ |
| GOTERM_MF_FAT | GO:0032559~Adenyl ribonucleotide binding | 6 | 19.4 | *NEK3, ACVR2B, CIT, DCK, P2RY1, TAP1* | 2.6 | 2.1 x 10^-2^ |
| GOTERM_MF_FAT | GO:0030554~Adenyl nucleotide binding | 6 | 19.4 | *NEK3, ACVR2B, CIT, DCK, P2RY1, TAP1* | 2.6 | 2.2 x 10^-2^ |
| GOTERM_BP_FAT | GO:0046903~Secretion | 5 | 16.1 | *VPS52, ACVR2B, F13A1, LMF1, P2RY1* | 3.0 | 2.2 x 10^-2^ |
| GOTERM_BP_FAT | GO:0046903~Positive regulation of catalytic activity | 6 | 19.4 | *DENND5A, ACVR2B, LMF1, MYL3, PSMB9, ROBO1* | 2.6 | 2.5 x 10^-2^ |
| GOTERM_MF_FAT | GO:0005198~Structural molecule activity | 4 | 12.9 | *MYOM2, MYL3, NPHP4, RPS18* | 3.4 | 2.7 x 10^-2^ |
| GOTERM_BP_FAT | GO:0030182~Neuron differentiation | 5 | 16.1 | *NEK3, GP5, NPHP4, NRL, ROBO1* | 2.7 | 3.3 x 10^-2^ |
| GOTERM_BP_FAT | GO:0007399~Nervous system development | 7 | 22.6 | *NEK3, CIT, GP5, NPHP4, NRL, P2RY1, ROBO1* | 2.1 | 3.6 x 10^-2^ |
| GOTERM_CC_FAT | GO:0005887~integral component of plasma membrane | 6 | 19.4 | *ACVR2B, GP5, P2RY1, ROBO1, SLC5A8, TAP1* | 2.3 | 3.8 x 10^-2^ |
| GOTERM_BP_FAT | GO:0016310~Phosphorylation | 7 | 22.6 | *NEK3, ACVR2B, CIT, DCK, MICAL1, PSMB9, P2RY1* | 2.1 | 4.1 x 10^-2^ |

Results present biological pathways enriched for the identified genes. Pathways are based on the Gene Ontology database implemented in DAVID(1). BP: Biological Process; CC: Cellular Component; FAT: filters out very broad GO terms based on a measured specificity of each term (not level-specificity); GO: Gene Ontology; MF: molecular function. Count: number of genes enriching the respective Gene Ontology term. %: the percentage of the identified genes for all genes known for the respective Gene Ontology term. Fold Enrichment: the x-fold enrichment of the respective Gene Ontology term by identified genes. Fisher Exact: p-value for the enrichment of Gene Ontology terms by the identified genes p-value <0.05.

**Table S10 Significantly enriched GO terms among CpGs with p-values <1.0 x 10^-4^ Identified in Child Peripheral Blood Associated with Liver Fat Accumulation in Childhood**

| Category | Term | Count | % | Genes | Fold enrichment | Fisher Exact |
| --- | --- | --- | --- | --- | --- | --- |
| GOTERM_BP_FAT | GO:0032506~Cytokinetic process | 3 | 4.5 | *BIN3, KIF20B, SPAST* | 62.6 | 1.3 x 10^-5^ |
| GOTERM_BP_FAT | GO:0021615~Glossopharyngeal nerve morphogenesis | 2 | 3.0 | *HOXA3, HOXD3* | 146.1 | 6.9 x 10^-5^ |
| GOTERM_BP_FAT | GO:1902410~Mitotic cytokinetic process | 2 | 3.0 | *BIN3, KIF20B* | 146.1 | 6.9 x 10^-5^ |
| GOTERM_BP_FAT | GO:0030878~Thyroid gland development | 3 | 4.5 | *FGF10, HOXA3, HOXD3* | 33.7 | 9.4 x 10^-5^ |
| GOTERM_BP_FAT | GO:0021563~Glossopharyngeal nerve development | 2 | 3.0 | *HOXA3, HOXD3* | 116.8 | 1.1 x 10^-4^ |
| GOTERM_BP_FAT | GO:0000281~Mitotic cytokinesis | 3 | 4.5 | *BIN3, KIF20B, SPAST* | 26.6 | 1.9 x 10^-4^ |
| GOTERM_BP_FAT | GO:0032467~Positive regulation of cytokinesis | 3 | 4.5 | *CXCR5, KIF20B, SPAST* | 23.7 | 2.7 x 10^-4^ |
| GOTERM_BP_FAT | GO:0061640~Cytoskeleton-dependent cytokinesis | 3 | 4.5 | *BIN3, KIF20B, SPAST* | 21.4 | 3.7 x 10^-4^ |
| GOTERM_BP_FAT | GO:0009952~Anterior/posterior pattern specification | 5 | 7.5 | *ALX4, HOXA3, HOXC13, HOXD3, SFRP1* | 7.3 | 6.0 x 10^-4^ |
| GOTERM_BP_FAT | GO:0007389~Pattern specification process | 7 | 10.4 | *ALX4, FGF10, HOXA3, HOXC13, HOXD3, SFRP1, SYNGAP1* | 4.7 | 6.9 x 10^-4^ |
| GOTERM_BP_FAT | GO:0000910~Cytokinesis | 4 | 6.0 | *CXCR5, BIN3, KIF20B, SPAST* | 9.7 | 7.5 x 10^-4^ |
| GOTERM_BP_FAT | GO:0048705~Skeletal system morphogenesis | 5 | 7.5 | *ALX4, COL13A1, HOXA3, HOXD3, SFRP1* | 6.9 | 7.6 x 10^-4^ |
| GOTERM_BP_FAT | GO:0032355~Response to estradiol | 4 | 6.0 | *DHH, FGF10, HSF1, SFRP1* | 9.7 | 7.8 x 10^-4^ |
| GOTERM_BP_FAT | GO:0003002~Regionalization | 6 | 9.0 | *ALX4, FGF10, HOXA3, HOXC13, HOXD3, SFRP1* | 5.3 | 8.8 x 10^-4^ |
| GOTERM_BP_FAT | GO:0032465~Regulation of cytokinesis | 3 | 4.5 | *CXCR5, KIF20B, SPAST* | 13.3 | 1.5 x 10^-3^ |
| GOTERM_MF_FAT | GO:0071837~HMG box domain binding | 2 | 3.0 | *ALX4, HOXA3* | 33.5 | 1.6 x 10^-3^ |
| GOTERM_BP_FAT | GO:0009887~Organ morphogenesis | 10 | 14.9 | *ALX4, CTNNA2, COL13A1, FGF10, HOXA3, HOXC13, HOXD3, SFRP1, SDC1, ZFPM1* | 2.9 | 1.8 x 10^-3^ |
| GOTERM_BP_FAT | GO:0046661~Male sex differentiation | 4 | 6.0 | *DHH, FGF10, SFRP1, SDC1* | 7.4 | 2.1 x 10^-3^ |
| GOTERM_BP_FAT | GO:0022405~Hair cycle process | 3 | 4.5 | *ALX4, FGF10, HOXC13* | 11.1 | 2.5 x 10^-3^ |
| GOTERM_BP_FAT | GO:0022404~Molting cycle process | 3 | 4.5 | *ALX4, FGF10, HOXC13* | 11.1 | 2.5 x 10^-3^ |
| GOTERM_BP_FAT | GO:0001942~Hair follicle development | 3 | 4.5 | *ALX4, FGF10, HOXC13* | 11.1 | 2.5 x 10^-3^ |
| GOTERM_BP_FAT | GO:0098773~Skin epidermis development | 3 | 4.5 | *ALX4, FGF10, HOXC13* | 10.8 | 2.7 x 10^-3^ |
| GOTERM_BP_FAT | GO:0051781~Positive regulation of cell division | 3 | 4.5 | *CXCR5, KIF20B, SPAST* | 10.6 | 2.9 x 10^-3^ |
| GOTERM_BP_FAT | GO:0048568~Embryonic organ development | 6 | 9.0 | *ALX4, FGF10, HSF1, HOXA3, HOXD3, ZFPM1* | 4.2 | 2.9 x 10^-3^ |
| GOTERM_BP_FAT | GO:0060740~Prostate gland epithelium morphogenesis | 2 | 3.0 | *FGF10, SFRP1* | 23.4 | 3.3 x 10^-3^ |
| GOTERM_BP_FAT | GO:0021602~Cranial nerve morphogenesis | 2 | 3.0 | *HOXA3, HOXD3* | 22.5 | 3.5 x 10^-3^ |
| GOTERM_BP_FAT | GO:0060606~Tube closure | 3 | 4.5 | *FGF10, KIF20B, SFRP1* | 9.7 | 3.6 x 10^-3^ |
| GOTERM_BP_FAT | GO:0060512~Prostate gland morphogenesis | 2 | 3.0 | *FGF10, SFRP1* | 21.6 | 3.8 x 10^-3^ |
| GOTERM_BP_FAT | GO:0048704~Embryonic skeletal system morphogenesis | 3 | 4.5 | *ALX4, HOXA3, HOXD3* | 9.4 | 4.0 x 10^-3^ |
| GOTERM_BP_FAT | GO:0010564~Regulation of cell cycle process | 7 | 10.4 | *CXCR5, GPR132, FGF10, KIF20B, PLK2, SFRP1, SPAST* | 3.4 | 4.0 x 10^-3^ |
| GOTERM_BP_FAT | GO:0006084~Acetyl-CoA metabolic process | 2 | 3.0 | *ACSS1, FASN* | 20.1 | 4.4 x 10^-3^ |
| GOTERM_BP_FAT | GO:0003401~Axis elongation | 2 | 3.0 | *FGF10, SFRP1* | 20.1 | 4.4 x 10^-3^ |
| GOTERM_BP_FAT | GO:0042303~Molting cycle | 3 | 4.5 | *ALX4, FGF10, HOXC13* | 9.0 | 4.5 x 10^-3^ |
| GOTERM_BP_FAT | GO:0042633~Hair cycle | 3 | 4.5 | *ALX4, FGF10, HOXC13* | 9.0 | 4.5 x 10^-3^ |
| GOTERM_CC_FAT | GO:0043198~Dendritic shaft | 2 | 3.0 | *GRM7, SYNGAP1* | 18.9 | 5.0 x 10^-3^ |
| GOTERM_BP_FAT | GO:0071392~Cellular response to estradiol stimulus | 2 | 3.0 | *HSF1, SFRP1* | 18.8 | 5.0 x 10^-3^ |
| GOTERM_BP_FAT | GO:0001823~Mesonephros development | 3 | 4.5 | *FGF10, SFRP1, SDC1* | 8.5 | 5.3 x 10^-3^ |
| GOTERM_BP_FAT | GO:0048468~Cell development | 14 | 20.9 | *BIN3, CTNNA2, DHH, DPYSL4, HOXD3, KIF20B, LINGO1, PIWIL1, PLK2, SFRP1, SPAST, SYNGAP1, SDC1, ZFPM1* | 2.1 | 5.6 x 10^-3^ |
| GOTERM_BP_FAT | GO:0045787~Positive regulation of cell cycle | 5 | 7.5 | *CXCR5, FGF10, KIF20B, PLK2, SPAST* | 4.3 | 6.0 x 10^-3^ |
| GOTERM_BP_FAT | GO:0001501~Skeletal system development | 6 | 9.0 | *ALX4, COL13A1, HOXA3, HOXD3, SFRP1, ZFPM1* | 3.6 | 6.4 x 10^-3^ |
| GOTERM_CC_FAT | GO:0098794~Postsynapse | 5 | 7.5 | *CTNNA2, COL13A1, GRM7, LRRTM1, SYNGAP1* | 4.0 | 7.8 x 10^-3^ |
| GOTERM_BP_FAT | GO:0010771~Negative regulation of cell morphogenesis involved in differentiation | 3 | 4.5 | *LINGO1, SFRP1, SYNGAP1* | 7.4 | 7.9 x 10^-3^ |
| GOTERM_CC_FAT | GO:0031226~Intrinsic component of plasma membrane | 12 | 17.9 | *CXCR5, C1QTNF1, NOXO1, COL13A1, GRM7, HLA-A, KCNK18, PTPRS, SLCO6A1, SYNGAP1, SDC1, TPSG1* | 2.1 | 7.9 x 10^-3^ |
| GOTERM_BP_FAT | GO:0030182~Neuron differentiation | 10 | 14.9 | *CTNNA2, DPYSL4, HOXD3, KIF20B, LINGO1, PLK2, RTN1, SFRP1, SPAST, SYNGAP1* | 2.4 | 8.1 x 10^-3^ |
| GOTERM_BP_FAT | GO:0090263~Positive regulation of canonical Wnt signaling pathway | 3 | 4.5 | *FGF10, RNF220, SFRP1* | 7.2 | 8.2 x 10^-3^ |
| GOTERM_BP_FAT | GO:0048706~Embryonic skeletal system development | 3 | 4.5 | *ALX4, HOXA3, HOXD3* | 7.1 | 8.6 x 10^-3^ |
| GOTERM_BP_FAT | GO:0050803~Regulation of synapse structure or activity | 4 | 6.0 | *CTNNA2, LRRTM1, PLK2, SYNGAP1* | 4.9 | 8.8 x 10^-3^ |
| GOTERM_BP_FAT | GO:0022008~Neurogenesis | 11 | 16.4 | *CTNNA2, DPYSL4, FGF10, HOXD3, KIF20B, LINGO1, PLK2, RTN1, SFRP1, SPAST, SYNGAP1* | 2.2 | 9.1 x 10^-3^ |
| GOTERM_BP_FAT | GO:0010977~Negative regulation of neuron projection development | 3 | 4.5 | *LINGO1, PLK2, SYNGAP1* | 6.7 | 1.0 x 10^-2^ |
| GOTERM_BP_FAT | GO:0035270~Endocrine system development | 3 | 4.5 | *FGF10, HOXA3, HOXD3* | 6.7 | 1.0 x 10^-2^ |
| GOTERM_BP_FAT | GO:0048812~Neuron projection morphogenesis | 6 | 9.0 | *CTNNA2, DPYSL4, KIF20B, LINGO1, SPAST, SYNGAP1* | 3.2 | 1.0 x 10^-2^ |
| GOTERM_BP_FAT | GO:0051302~Regulation of cell division | 3 | 4.5 | *CXCR5, KIF20B, SPAST* | 6.6 | 1.1 x 10^-2^ |
| GOTERM_BP_FAT | GO:0048565~Digestive tract development | 3 | 4.5 | *ALX4, FGF10, SFRP1* | 6.6 | 1.1 x 10^-2^ |
| GOTERM_BP_FAT | GO:0090068~Positive regulation of cell cycle process | 4 | 6.0 | *CXCR5, KIF20B, PLK2, SPAST* | 4.7 | 1.1 x 10^-2^ |
| GOTERM_BP_FAT | GO:0046546~Development of primary male sexual characteristics | 3 | 4.5 | *DHH, SFRP1, SDC1* | 6.4 | 1.1 x 10^-2^ |
| GOTERM_BP_FAT | GO:0008584~Male gonad development | 3 | 4.5 | *DHH, SFRP1, SDC1* | 6.4 | 1.1 x 10^-2^ |
| GOTERM_BP_FAT | GO:0048167~Regulation of synaptic plasticity | 3 | 4.5 | *LRRTM1, PLK2, SYNGAP1* | 6.3 | 1.2 x 10^-2^ |
| GOTERM_CC_FAT | GO:0030424~Axon | 5 | 7.5 | *CTNNA2, GRM7, KIF20B, LRRTM1, SPAST* | 3.6 | 1.2 x 10^-2^ |
| GOTERM_BP_FAT | GO:0035148~Tube formation | 3 | 4.5 | *FGF10, KIF20B, SFRP1* | 6.2 | 1.3 x 10^-2^ |
| GOTERM_BP_FAT | GO:0007267~Cell-cell signaling | 11 | 16.4 | *C1QTNF1, DHH, FGF10, GRM7, LRRTM1, PLK2, PTPRS, RNF220, SFRP1, SYNGAP1, SDC1* | 2.1 | 1.3 x 10^-2^ |
| GOTERM_BP_FAT | GO:0007399~Nervous system development | 14 | 20.9 | *CTNNA2, DHH, DPYSL4, FGF10, HOXA3, HOXD3, KIF20B, LINGO1, LRRTM1, PLK2, RTN1, SFRP1, SPAST, SYNGAP1* | 1.9 | 1.3 x 10^-2^ |
| GOTERM_BP_FAT | GO:0048598~Embryonic morphogenesis | 6 | 9.0 | *ALX4, FGF10, HOXA3, HOXD3, KIF20B, SFRP1* | 3.1 | 1.3 x 10^-2^ |
| GOTERM_BP_FAT | GO:0048771~Tissue remodeling | 3 | 4.5 | *FGF10, HOXA3, SFRP1* | 6.1 | 1.3 x 10^-2^ |
| GOTERM_BP_FAT | GO:0055123~Digestive system development | 3 | 4.5 | *ALX4, FGF10, SFRP1* | 6.0 | 1.3 x 10^-2^ |
| GOTERM_BP_FAT | GO:0007548~Sex differentiation | 4 | 6.0 | *DHH, FGF10, SFRP1, SDC1* | 4.3 | 1.3 x 10^-2^ |
| GOTERM_BP_FAT | GO:0043009~Chordate embryonic development | 6 | 9.0 | *ALX4, HSF1, HOXA3, HOXD3, KIF20B, SFRP1* | 3.0 | 1.4 x 10^-2^ |
| GOTERM_BP_FAT | GO:0048608~Reproductive structure development | 5 | 7.5 | *DHH, FGF10, HSF1, SFRP1, SDC1* | 3.5 | 1.4 x 10^-2^ |
| GOTERM_BP_FAT | GO:0009792~Embryo development ending in birth or egg hatching | 6 | 9.0 | *ALX4, HSF1, HOXA3, HOXD3, KIF20B, SFRP1* | 3.0 | 1.4 x 10^-2^ |
| GOTERM_BP_FAT | GO:0048732~Gland development | 5 | 7.5 | *FASN, FGF10, HOXA3, HOXD3, SFRP1* | 3.5 | 1.4 x 10^-2^ |
| GOTERM_BP_FAT | GO:0016331~Morphogenesis of embryonic epithelium | 3 | 4.5 | *FGF10, KIF20B, SFRP1* | 5.9 | 1.4 x 10^-2^ |
| GOTERM_BP_FAT | GO:0061458~Reproductive system development | 5 | 7.5 | *DHH, FGF10, HSF1, SFRP1, SDC1* | 3.5 | 1.5 x 10^-2^ |
| GOTERM_BP_FAT | GO:0061061~Muscle structure development | 6 | 9.0 | *ALX4, BIN3, CTNNA2, FGF10, SDC1, ZFPM1* | 3.0 | 1.5 x 10^-2^ |
| GOTERM_BP_FAT | GO:0009790~Embryo development | 8 | 11.9 | *ALX4, FGF10, HSF1, HOXA3, HOXD3, KIF20B, SFRP1, ZFPM1* | 2.4 | 1.5 x 10^-2^ |
| GOTERM_BP_FAT | GO:0050804~Modulation of chemical synaptic transmission | 4 | 6.0 | *GRM7, LRRTM1, PLK2, SYNGAP1* | 4.2 | 1.5 x 10^-2^ |
| GOTERM_BP_FAT | GO:0051960~Regulation of nervous system development | 7 | 10.4 | *HOXD3, KIF20B, LINGO1, LRRTM1, PLK2, SFRP1, SYNGAP1* | 2.7 | 1.5 x 10^-2^ |
| GOTERM_BP_FAT | GO:0031345~Negative regulation of cell projection organization | 3 | 4.5 | *LINGO1, PLK2, SYNGAP1* | 5.7 | 1.6 x 10^-2^ |
| GOTERM_BP_FAT | GO:0030177~Positive regulation of Wnt signaling pathway | 3 | 4.5 | *FGF10, RNF220, SFRP1* | 5.7 | 1.6 x 10^-2^ |
| GOTERM_MF_FAT | GO:0008201~Heparin binding | 3 | 4.5 | *COL13A1, FGF10, SFRP1* | 5.7 | 1.6 x 10^-2^ |
| GOTERM_BP_FAT | GO:0048699~Generation of neurons | 10 | 14.9 | *CTNNA2, DPYSL4, HOXD3, KIF20B, LINGO1, PLK2, RTN1, SFRP1, SPAST, SYNGAP1* | 2.2 | 1.6 x 10^-2^ |
| GOTERM_CC_FAT | GO:0005887~Integral component of plasma membrane | 11 | 16.4 | *CXCR5, C1QTNF1, NOXO1, COL13A1, GRM7, HLA-A, KCNK18, PTPRS, SLCO6A1, SDC1, TPSG1* | 2.0 | 1.6 x 10^-2^ |
| GOTERM_BP_FAT | GO:0060070~Canonical Wnt signaling pathway | 4 | 6.0 | *FGF10, RNF220, SFRP1, SDC1* | 4.1 | 1.6 x 10^-2^ |
| GOTERM_BP_FAT | GO:0048562~Embryonic organ morphogenesis | 4 | 6.0 | *ALX4, FGF10, HOXA3, HOXD3* | 4.1 | 1.7 x 10^-2^ |
| GOTERM_BP_FAT | GO:0051726~Regulation of cell cycle | 8 | 11.9 | *CXCR5, GPR132, BOP1, FGF10, KIF20B, PLK2, SFRP1, SPAST* | 2.4 | 1.7 x 10^-2^ |
| GOTERM_BP_FAT | GO:0051129~Negative regulation of cellular component organization | 6 | 9.0 | *HSF1, LINGO1, LRRTM1, PLK2, SFRP1, SYNGAP1* | 2.8 | 1.9 x 10^-2^ |
| GOTERM_CC_FAT | GO:0005911~Cell-cell junction | 6 | 9.0 | *FRMD4A, CTNNA2, CLDN14, COL13A1, EPN2, FASN* | 2.8 | 1.9 x 10^-2^ |
| GOTERM_BP_FAT | GO:0010721~Negative regulation of cell development | 4 | 6.0 | *LINGO1, PLK2, SFRP1, SYNGAP1* | 3.8 | 2.1 x 10^-2^ |
| GOTERM_BP_FAT | GO:0031175~Neuron projection development | 7 | 10.4 | *CTNNA2, DPYSL4, KIF20B, LINGO1, PLK2, SPAST, SYNGAP1* | 2.5 | 2.1 x 10^-2^ |
| GOTERM_BP_FAT | GO:0043066~Negative regulation of apoptotic process | 7 | 10.4 | *TNFAIP8, FGF10, HSF1, PLK2, SFRP1, SYNGAP1, ZC3HC1* | 2.5 | 2.1 x 10^-2^ |
| GOTERM_BP_FAT | GO:0003006~Developmental process involved in reproduction | 6 | 9.0 | *DHH, FGF10, HSF1, PIWIL1, SFRP1, SDC1* | 2.8 | 2.1 x 10^-2^ |
| GOTERM_BP_FAT | GO:0045596~Negative regulation of cell differentiation | 6 | 9.0 | *FGF10, LINGO1, PLK2, SFRP1, SYNGAP1, ZFPM1* | 2.7 | 2.2 x 10^-2^ |
| GOTERM_BP_FAT | GO:0043069~Negative regulation of programmed cell death | 7 | 10.4 | *TNFAIP8, FGF10, HSF1, PLK2, SFRP1, SYNGAP1, ZC3HC1* | 2.5 | 2.2 x 10^-2^ |
| GOTERM_BP_FAT | GO:0000902~Cell morphogenesis | 9 | 13.4 | *BIN3, CTNNA2, DPYSL4, KIF20B, LINGO1, SFRP1, SPAST, SYNGAP1, ZFPM1* | 2.1 | 2.4 x 10^-2^ |
| GOTERM_BP_FAT | GO:0007049~Cell cycle | 11 | 16.4 | *CXCR5, GPR132, BIN3, FGF10, HSF1, KIF20B, PIWIL1, PLK2, SFRP1, SPAST, ZC3HC1* | 1.9 | 2.5 x 10^-2^ |
| GOTERM_BP_FAT | GO:0007155~Cell adhesion | 11 | 16.4 | *C1QTNF1, CTNNA2, CLDN14, COL13A1, EPN2, FASN, HOXD3, HLA-A, PTPRS, SFRP1, ZFPM1* | 1.9 | 2.7 x 10^-2^ |
| GOTERM_BP_FAT | GO:0022610~Biological adhesion | 11 | 16.4 | *C1QTNF1, CTNNA2, CLDN14, COL13A1, EPN2, FASN, HOXD3, HLA-A, PTPRS, SFRP1, ZFPM1* | 1.9 | 2.8 x 10^-2^ |
| GOTERM_BP_FAT | GO:0050767~Regulation of neurogenesis | 6 | 9.0 | *HOXD3, KIF20B, LINGO1, PLK2, SFRP1, SYNGAP1* | 2.6 | 2.9 x 10^-2^ |
| GOTERM_BP_FAT | GO:1903047~Mitotic cell cycle process | 7 | 10.4 | *GPR132, BIN3, FGF10, KIF20B, PLK2, SPAST, ZC3HC1* | 2.3 | 3.1 x 10^-2^ |
| GOTERM_BP_FAT | GO:0030030~Cell projection organization | 9 | 13.4 | *BIN3, CTNNA2, DPYSL4, KIF20B, LINGO1, PLK2, SFRP1, SPAST, SYNGAP1* | 2.0 | 3.2 x 10^-2^ |
| GOTERM_BP_FAT | GO:0060548~Negative regulation of cell death | 7 | 10.4 | *TNFAIP8, FGF10, HSF1, PLK2, SFRP1, SYNGAP1, ZC3HC1* | 2.3 | 3.3 x 10^-2^ |
| GOTERM_BP_FAT | GO:0032989~Cellular component morphogenesis | 9 | 13.4 | *BIN3, CTNNA2, DPYSL4, KIF20B, LINGO1, SFRP1, SPAST, SYNGAP1, ZFPM1* | 2.0 | 3.4 x 10^-2^ |
| GOTERM_CC_FAT | GO:0043005~Neuron projection | 7 | 10.4 | *CTNNA2, GRM7, KIF20B, LRRTM1, PLK2, SPAST, SYNGAP1* | 2.2 | 3.8 x 10^-2^ |
| GOTERM_CC_FAT | GO:0009986~Cell surface | 6 | 9.0 | *CXCR5, FGF10, LRRTM1, HLA-A, SFRP1, SDC1* | 2.4 | 3.9 x 10^-2^ |
| GOTERM_BP_FAT | GO:0022402~Cell cycle process | 9 | 13.4 | *CXCR5, GPR132, BIN3, FGF10, HSF1, KIF20B, PLK2, SPAST, ZC3HC1* | 1.9 | 4.0 x 10^-2^ |
| GOTERM_BP_FAT | GO:0048585~Negative regulation of response to stimulus | 9 | 13.4 | *C1QTNF1, SEC14L1, CTNNA2, FGF10, LRRTM1, HLA-A, SFRP1, SYNGAP1, ZC3HC1* | 1.9 | 4.2 x 10^-2^ |
| GOTERM_MF_FAT | GO:0005515~Protein binding | 36 | 53.7 | *ATP11A, CXCR5, C1QTNF1, MXD3, NOXO1, SEC14L1, TNFAIP8, ACSS1, BOP1, BIN3, CTNNA2, COL13A1, DHH, DPYSL4, EPN2, FASN, FGF10, HSF1, HOXA3, HOXC13, HOXD3, KIF20B, LINGO1, HLA-A, PIWIL1, PLK2, PSG5, PTPRS, RTN1, RNF220, SFRP1, SPAST, SDC1, ZC3HC1, ZNF783, ZFPM1* | 1.2 | 4.3 x 10^-2^ |
| GOTERM_BP_FAT | GO:0098609~Cell-cell adhesion | 8 | 11.9 | *C1QTNF1, CTNNA2, CLDN14, COL13A1, EPN2, FASN, HLA-A, ZFPM1* | 2.0 | 4.4 x 10^-2^ |

Results present biological pathways enriched for the identified genes. Pathways are based on the Gene Ontology database implemented in DAVID(1). BP: Biological Process; CC: Cellular Component; FAT: filters out very broad GO terms based on a measured specificity of each term (not level-specificity); GO: Gene Ontology; MF: molecular function. Count: number of genes enriching the respective Gene Ontology term. %: the percentage of the identified genes for all genes known for the respective Gene Ontology term. Fold Enrichment: the x-fold enrichment of the respective Gene Ontology term by identified genes. Fisher Exact: p-value for the enrichment of Gene Ontology terms by the identified genes p-value <0.05.

**Reference**

1. Huang da W, Sherman BT, Lempicki RA. Systematic and integrative analysis of large gene lists using DAVID bioinformatics resources. Nat Protoc. 2009;4(1):44-57.
